# Competitive coordination strategy for the synthesis of hierarchical-pore metal–organic framework nanostructures†
†Electronic supplementary information (ESI) available. See DOI: 10.1039/c6sc02272c



**DOI:** 10.1039/c6sc02272c

**Published:** 2016-08-05

**Authors:** Su He, Yifeng Chen, Zhicheng Zhang, Bing Ni, Wei He, Xun Wang

**Affiliations:** a Department of Chemistry , Tsinghua University , Beijing , 100084 , China . Email: wangxun@mail.tsinghua.edu.cn; b School of Pharmaceutical Science , Tsinghua University , Beijing , 100084 , China

## Abstract

Metal–organic frameworks (MOFs) usually have micropores smaller than 2 nm, which may restrict their applications in some cases. Hierarchical-pore MOFs (H-MOFs) are a new family of MOF materials, possessing both micro- and mesopores to address this problem. Here we demonstrate a competitive coordination strategy for the synthesis of H-MOF nanostructures, such as two-dimensional (2D) H-MOF nanosheets and H-MOF nanocubes, evolving through an etching process tuned by a competitive ligand. The as-synthesized 2D H-MOF nanosheets can serve as a substrate to *in situ* immobilize Pd nanoparticles to achieve a surfactant-free Pd catalyst, by means of a simple soaking method of Pd^2+^ precursors. Combined with the unique structure and gas adsorption capacity of H-MOF-5, the Pd-H-MOF-5 catalyst exhibits superior catalytic performance.

## Abstract 2

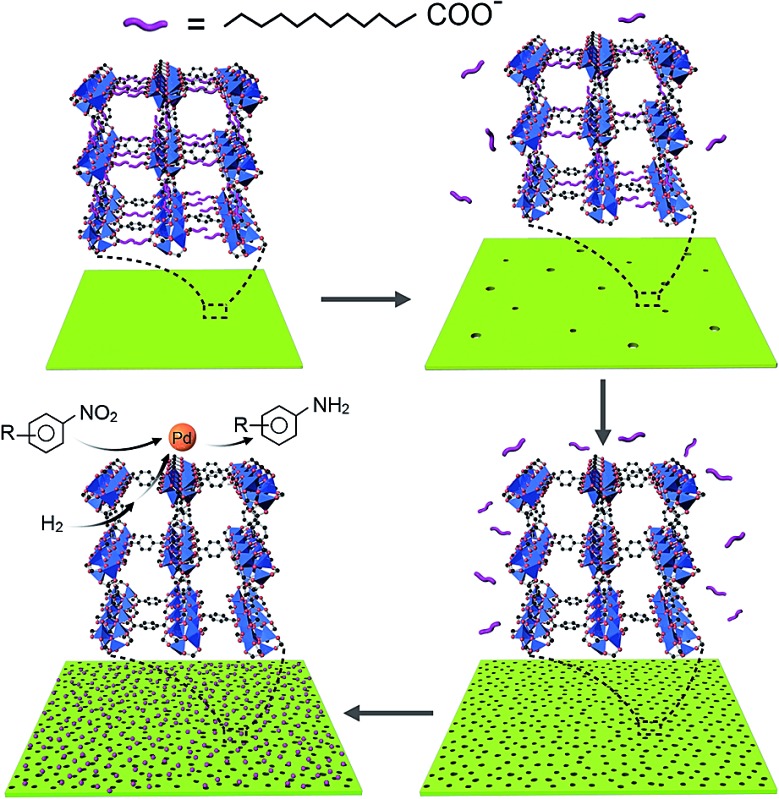
We demonstrate a competitive coordination strategy for the synthesis of H-MOF nanostructures, such as two-dimensional H-MOF nanosheets and H-MOF nanocubes, evolving through an etching process tuned by a competitive ligand.

## Introduction

Constructed with inorganic nodes and organic linkers with two or more ligation sites, metal–organic frameworks (MOFs) have received tremendous attention in recent years.^1–3^ Their attractive diversity in structure could be tailored by rational design of metal-based inorganic nodes and organic linkers with judicious geometrical and functional selections. Traditional structural studies of MOFs have long since developed, with more and more new MOFs burgeoning,^4,5^ and they have shown widespread potential applications in gas storage,^6^ adsorption,^7,8^ molecular separation,^9^ catalysis,^10–12^ imaging,^13^ and so on. Notably, two-dimensional (2D) MOFs, with many highly active sites on their surfaces like other 2D materials,^14–16^ have attracted increasing interest recently.^17–19^ Besides, due to their large surface areas, guest molecules could effectively pass through the micropores,^18^ which could benefit gas-involved catalytic reactions.

Meanwhile, MOFs, owing to their microporosity (pore size < 2 nm), benefit the adsorption and separation of small molecules. However, the small pore size in microporous MOFs in turn limits fast mass diffusion and restrains larger molecules from entering into the center through the MOF micropores, hence restricting the potential practical applications in some cases.^20,21^ Therefore, enlarging the pore size or creating some larger pores in primitive MOF structures is necessary, however challenging at present. Once introducing mesopores into the previously microporous MOF structures, the as-achieved hierarchical-pore MOFs (H-MOFs) would be advantageous in complete utilization of materials.^22,23^ The micropores provide exceptionally high surface areas and large pore volumes, whereas the mesopores facilitate diffusion and accessibility. Many efforts have been made to reach this aim. The most commonly adopted one is the ligand-extension method^24^ – by using longer organic ligands to achieve the mesoporosity – and quite a few MOFs with ultralarge pores have been elaborately designed and synthesized.^25^ However, it is usually limited by instability and possible interpenetration as the size extends, along with the high cost and difficulty in synthesizing larger organic ligands. Besides, the surfactant-templating method,^22^ microwave-assisted method,^26^ CO_2_-expanded liquid route,^27^ ionic liquid system,^28^ and post-synthetic method^29^ are also alternatives for creating mesopores. Nonetheless, these innovative methods require either a template or complicated techniques, and are difficult to extend to other systems. So new versatile methods are imperative to establish stable and high-quality H-MOFs.

Herein, we developed a competitive coordination strategy for synthesis of H-MOF nanostructures, possessing both micro- and mesopores in the network. The as-synthesized 2D H-MOF nanosheets and H-MOF nanocubes evolved through an etching process tuned by a competitive ligand as a modulator. Furthermore, the 2D H-MOF nanosheets could be used as a substrate to *in situ* form and immobilize Pd nanoparticles without any surfactant (denoted as Pd-H-MOF), by means of a simple soaking method of Pd^2+^ precursors. Taking advantage of the gas adsorption capacity of H-MOFs, the surfactant-free Pd catalyst exhibited excellent catalytic performance.

## Results and discussion

**Fig. 1:**
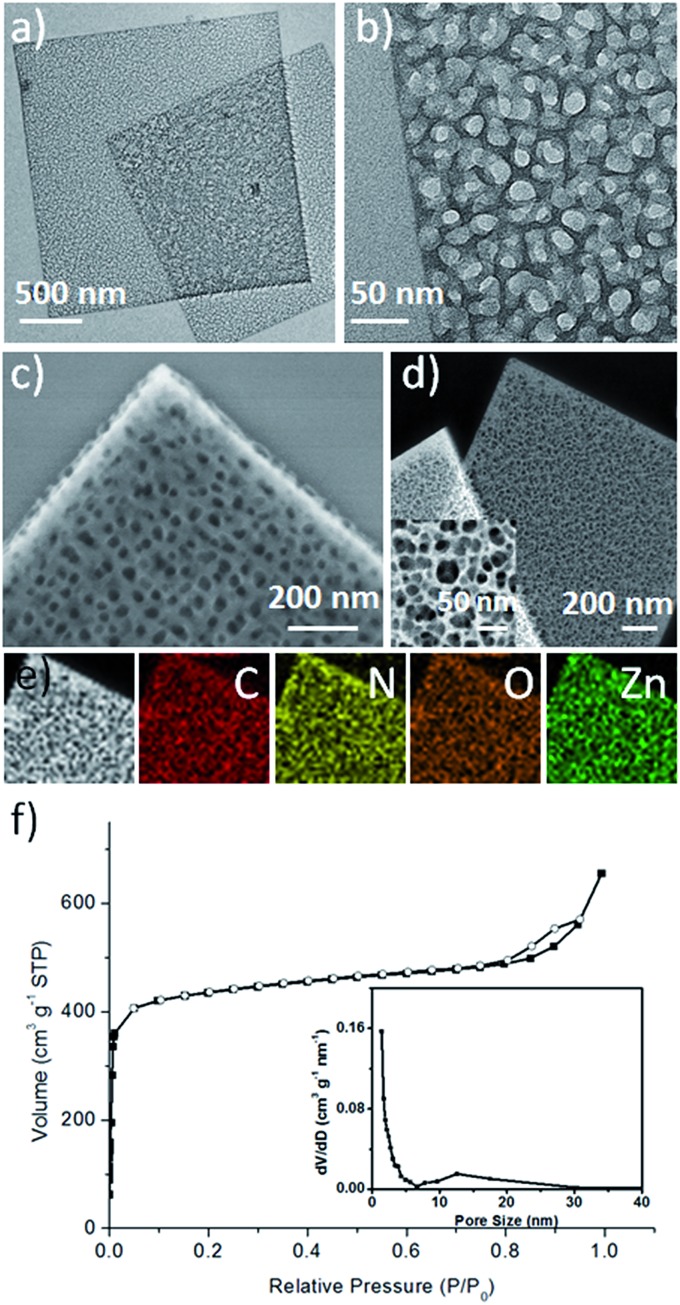
(a and b) TEM images, (c) SEM image, (d) HAADF-STEM images, (e) EDX elemental mapping images and (f) nitrogen adsorption (■) and desorption (○) isotherms measured at 77 K of 2D H-MOF-5 nanosheets. Inset in (f): the corresponding pore size distribution.

MOF-5 was first selected as an example to prove the concept of the competitive coordination strategy. The 2D H-MOF-5 nanosheets were synthesized *via* a simple solvothermal reaction of zinc acetate (Zn(OAc)_2_·2H_2_O), 1,4-benzenedicarboxylic acid (H_2_BDC), lauric acid (LA), and polyvinylpyrrolidone, K-30 (PVP, K-30) in *N*,*N*-dimethylacetamide (DMAC). The porous morphology and structure were identified by diverse characterizations. The transmission electron microscopy (TEM) images (Fig. 1a, b and S1†) show the typical shape of 2D H-MOF-5 nanosheets. All the nanosheets spread flatly without curls or wrinkles and the length and width could reach micron-scale. The porosity of the nanosheets was further confirmed more distinctly by the scanning electron microscopy (SEM) image (Fig. 1c) and high-angle annular dark-field scanning TEM (HAADF-STEM) image (Fig. 1d), where numerous mesopores are visible throughout the whole nanosheets, even on the edges. The high-magnification HAADF-STEM image (inset of Fig. 1d) provides access to better observe the mesopores, and demonstrates that the sizes of the pores inside are not so uniform and that the pores form a network of multilayers like a sponge rather than one single layer with pores on it like a paper-cut. The thickness of the 2D porous nanosheets was determined to be approximately 19 nm, using an atomic force microscope (AFM) (Fig. S2†).

As for the composition, elemental mapping by energy-dispersive X-ray spectroscopy (EDX) shows that the elements C, O, N, and Zn are uniformly distributed throughout the whole nanosheet (Fig. 1e). And the X-ray photoelectron spectrum (XPS) also identifies the composition (Fig. S3†), where the existence of N is attributed to the residue of solvent molecules and pyrrolidone rings of PVP. The Fourier transform infrared (FT-IR) spectrum proves that the coordinative interaction between Zn_4_O clusters and the carboxylic group of deprotonated H_2_BDC was accomplished, as disclosed by a red shift in the C=O stretching frequency from 1680 cm^–1^ – the characteristic C=O stretching frequency of uncoordinated free H_2_BDC^30^ – to 1603 cm^–1^ (Fig. S4†). The powder X-ray diffraction (PXRD) pattern of the product, which was obtained after evacuation of solvent molecules at 100 °C under vacuum for 6 h, corresponds well with that of the simulated MOF-5 XRD patterns^31^ (Fig. S5†), while the difference in relative peak intensities might originate from preferential orientation.

The 2D H-MOF-5 nanosheets exhibited high thermal stability (>400 °C) evidenced by the thermogravimetric (TG) curve (Fig. S6†) taken in air (nitrogen). The weight loss below 100 °C could be ascribed to the solvent liberation or the loss of guest molecules, while the significant weight loss in the range from 400 °C to 480 °C arises from the framework collapse.^20,31^ The nanosheets also showed permanent porosity after evacuation of solvent molecules. N_2_ sorption data at 77 K displayed a type I isotherm at relatively low *P*/*P*_0_ pressures for a micropore structure and a typical type IV isotherm at relatively high *P*/*P*_0_ (ref. 27) (Fig. 1f), which is related to the capillary condensation associated with mesopore channels,^32^ suggesting the additional presence of mesopores. The mesopore diameter distribution was calculated using the Barrett–Joyner–Halenda (BJH) method (inset of Fig. 1f), indicating a broad range of mesopore diameters, from 6 nm to 30 nm, which could also be confirmed visually by TEM and SEM images. The micropore diameter distribution was also calculated using the Saito–Foley (SF) method^32^ (Fig. S7†), revealing the existence of micropores, which are the inherent characteristic of the framework of MOF-5.^1^ Due to the hierarchically porous structures, the BET specific surface area of the sample could reach up to 1669 m^2^ g^–1^.

**Fig. 2:**
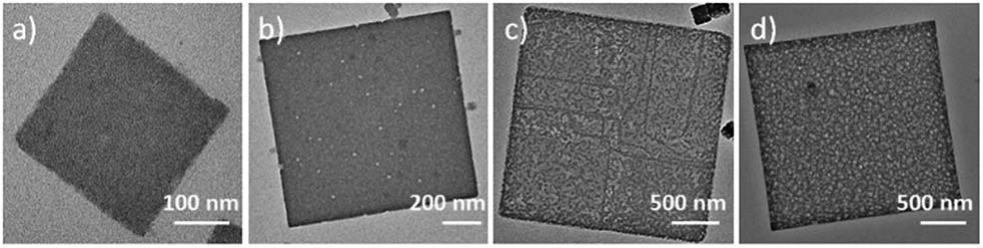
TEM images of the competitive coordination etching process. (a) Initial solid MOF-5 nanosheet, (b) intermediate I with several mesopores, (c) intermediate II with more mesopores, and (d) final 2D H-MOF-5 nanosheets.

The formation of these 2D H-MOF-5 nanosheets underwent a morphologic evolution. Small solid square nanosheets came into being at the early stage. Then an etching process took place, with more and more visible pores emerging both inside and on the edges, until they sprawled over the entire nanosheets (Fig. 2). The formation of MOF-5 was accomplished at the very beginning, as confirmed by PXRD (Fig. S8†) and FT-IR results (Fig. S9†), and no obvious compositional changes happened during the etching process.

**Fig. 3:**
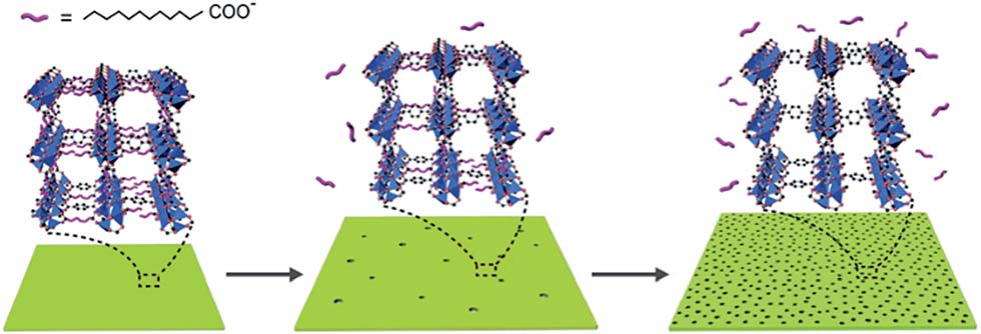
Schematic illustration of the competitive coordination etching process.

LA could coordinate *via* its carboxyl group to surface-exposed Zn_4_O sites, acting as a diffusion limiter for both the metal ions and organic ligands, hence controlling crystal growth.^7^ We proposed a competitive coordination mechanism to explain the evolutionary process, in which LA could serve as a competitive ligand or modulator in the coordination to Zn_4_O clusters.^33^ Deprotonated H_2_BDC and LA were both capable of coordinating to Zn_4_O clusters in the initial stage, though the coordination of deprotonated LA was much weaker, because LA has one single carboxyl group while H_2_BDC has two to form the stable MOF networks. As the reaction proceeded, the coordination of deprotonated H_2_BDC predominated, while the weakly bonded deprotonated LA to Zn_4_O clusters dissociated to release LA from the framework. The migration of LA could provide copious sites for further etching and afterwards enable the creation of mesopores in the nanosheets (Fig. 3).

To confirm the mechanism and disclose the details, a series of control experiments were conducted (Fig. S10–S12†). It turns out that the final morphology is sensitive to initial concentration and reaction temperature, so these parameters need to be reconsidered when extending to other MOFs. All the reactants are indispensable. Zn(OAc)_2_·2H_2_O acts as metallic nodes and H_2_BDC as organic linkers to construct the elementary framework of MOF-5. PVP determines the formation of 2D nanosheets, and the molecular weight of PVP plays an important role in directing the morphology (Fig. S13†). Meanwhile LA, as mentioned before, directs the etching process and mesoporous structure. Without LA, merely solid nanosheets could be prepared (Fig. S12a†), because no competitive coordination was involved to provide empty sites for the further etching process and the formal coordination led to complete structures. Notably, other long-chain organic acids with 10–18 carbon atoms could function as well (Fig. S14†) to achieve 2D H-MOF-5 nanosheets, whereas short-chain organic acids or alkanes could only result in solid nanosheets (Fig. S15†).

**Fig. 4:**
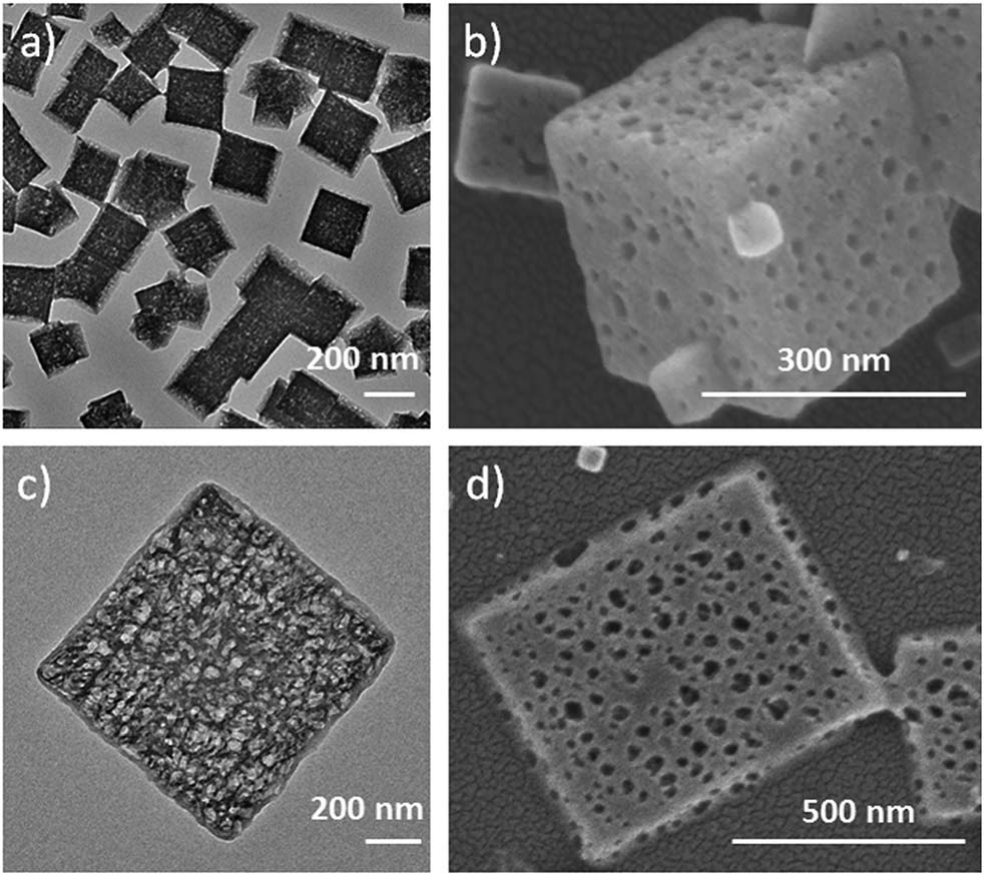
(a) TEM, and (b) SEM images of IRMOF-3 nanocages; (c) TEM, and (d) SEM images of hierarchical-pore 2D IRMOF-8 hierarchical-pore nanosheets.

This competitive coordination strategy is also effective in directing other H-MOFs. By expansion of the MOF-5 structure, other hierarchical-pore IRMOFs, an isoreticular series sharing the same topology with MOF-5, could likewise be achieved. For IRMOF-3, functionalized with amino groups, H-MOF nanocubes could be prepared through competitive coordination. And for IRMOF-8, extending from the original ligand of the one phenylene ring to two, 2D H-MOF nanosheets could be realized similar to the previous result of MOF-5 (Fig. 4). They were further characterized (Fig. S16 and S17†).

**Fig. 5:**
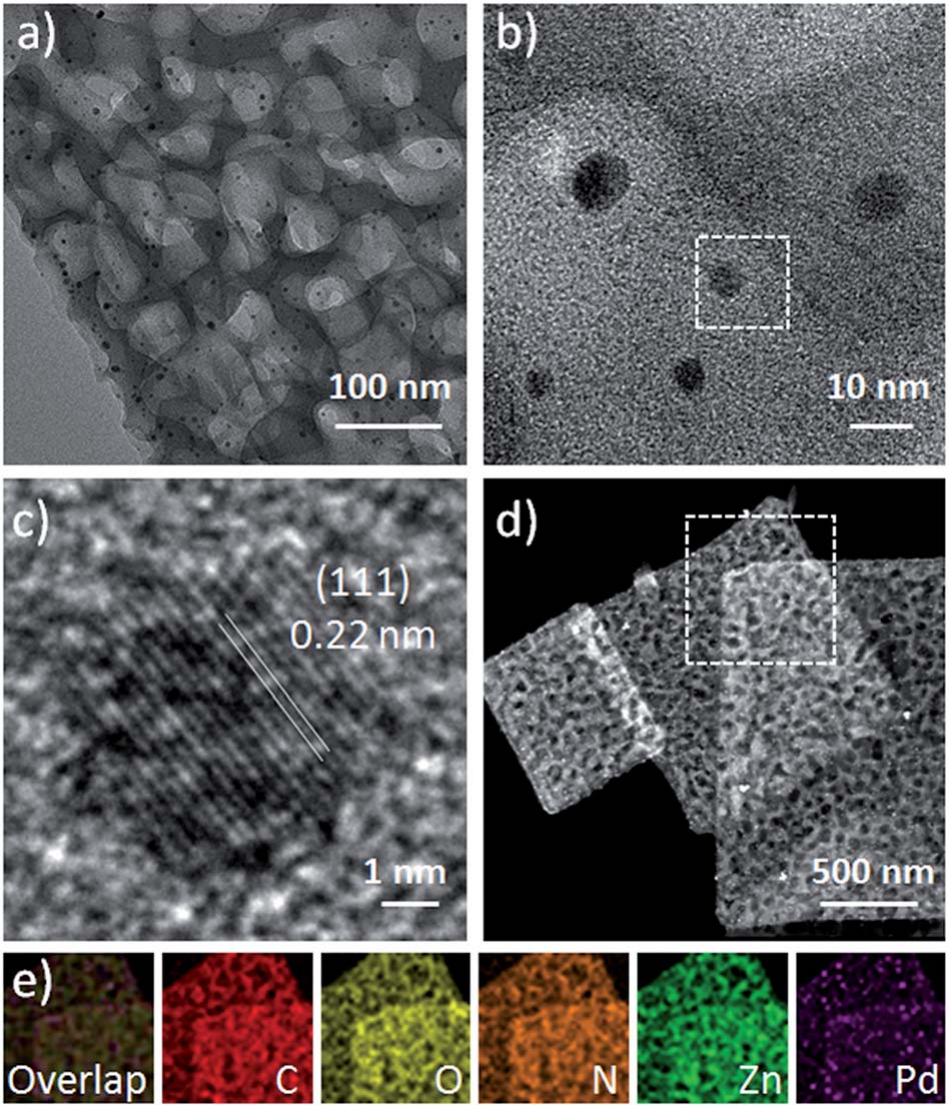
(a) TEM image, (b and c) HRTEM images, and (d) HAADF-STEM image of Pd-H-MOF-5 nanosheets. (e) EDX elemental mapping results of the selected area marked by the white dashed line in (d). (c) is the magnified HRTEM image of the selected area marked by the white dashed line in (b).

The 2D H-MOF-5 nanosheets can be used to confine the growth of surfactant-free Pd nanoparticles through a simple soaking method with a Pd^2+^ precursor in the mixed solvent of DMAC and EtOH. Up to now, many reports have discussed the loading of various noble metal nanoparticles into MOF nanostructures, which requires extra effort to synthesize noble metal nanoparticles alone in advance, where the introduction of surfactants is mostly difficult to avoid.^34^ In a typical experiment, we chose PdCl_2_ as the Pd^2+^ precursor. Due to the reducibility of DMAC, the reduction of Pd^2+^ was easy to achieve. Pd nanoparticles with a size of sub-10 nm were uniformly distributed in the nanosheets (Fig. 5d), without obvious agglomeration. Meanwhile, the 2D porous morphology and crystal structure of H-MOF-5 could be maintained, as demonstrated by PXRD results (Fig. S18†). The morphology, size and composition of the Pd-H-MOF-5 were also illustrated by TEM, HRTEM, EDX, and EDX mapping images and inductively coupled plasma optical emission spectrometry (ICP-OES) (Fig. 5 and S19†). Additionally, Pd acetylacetonate (Pd(acac)_2_) could also serve as the Pd^2+^ precursor to acquire similar results (Fig. S20†), although the reduction was much more difficult and required more soaking time. But no Pd nanoparticles could be obtained without 2D H-MOF-5 nanosheets in the mixed solvent of DMAC and EtOH, even after soaking for two days. This indicates that the nanosheets may serve as a substrate to provide appropriate nucleation sites for Pd nanoparticles to form and grow easily, and the solvent DMAC could effectively assist the reduction of Pd^2+^.

**Fig. 6:**
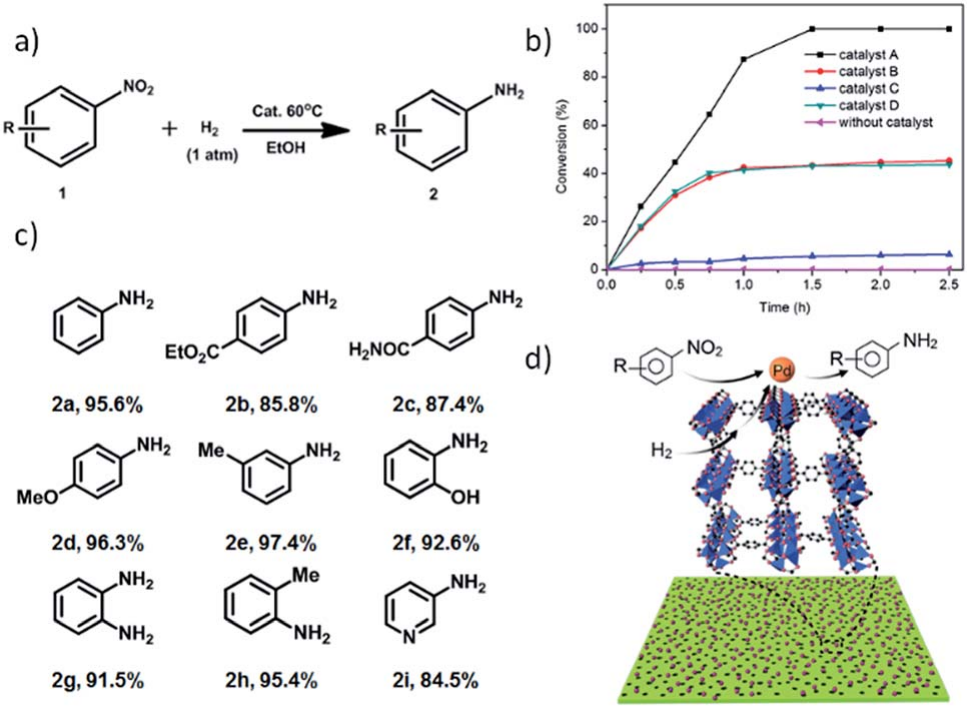
(a) Reduction of nitroarene. Reaction conditions: 1 (0.5 mmol), Pd-H-MOF-5 nanosheets (0.01 equiv.), H_2_ (balloon) in EtOH at 60 °C, GC yields. (b) Conversion (%) as a function of time in the reduction reaction over Pd-H-MOF-5 nanosheets (catalyst A), Pd-H-MOF-5 post-treated with H_2_O (catalyst B), Pd-H-MOF-5 nanosheets with surfactants (catalyst C), Pd@bulk MOF-5 (catalyst D) and without catalyst. (c) Products and yields of isolated products from the reduction of nitroarene catalyzed by Pd-H-MOF-5 nanosheets for 4 h. (d) Schematic representation of the reduction of nitroarene over Pd-H-MOF-5 nanosheets.

Taking into account the hydrogen storage capacity of MOF-5 (ref. 6) and the activity of surfactant-free Pd nanoparticles, we envisioned that the Pd-H-MOF nanosheets should integrate these advantages and show excellent catalytic performance. A Pd-catalyzed reduction of nitroarene using hydrogen as a reductant is an important industrial process (Fig. 6a), and was chosen to demonstrate the structural advantage. In order to confirm the effectiveness of Pd-H-MOF-5 nanosheets (catalyst A), three other kinds of Pd catalyst were synthesized as well for comparison (Fig. S21†). Catalyst B was Pd-H-MOF-5 nanosheets post-treated with water to destroy the MOF nanostructures, while the Pd nanoparticles maintained pristine structures. Catalyst C had a similar shape to Pd-H-MOF nanosheets, except for the synthetic procedure involving surfactants, to ascertain the virtue of surfactant-free Pd as catalysts. Catalyst D was bulk MOF-5 immobilized with Pd nanoparticles (Pd@bulk MOF-5) using the similar soaking method, to illustrate the benefit of H-MOF-5 nanosheets. Fig. 6b exhibits the kinetic curves for the reduction of nitroarene reactions on these four catalysts, where the excellent catalytic activity of catalyst A was uncovered. The results demonstrate the structural advantages of H-MOF-5 and surfactant-free Pd nanoparticles. In addition, substrates with different functional groups were likewise tested to obtain the corresponding products (Fig. 6c), with both electron-withdrawing (2b and 2c) and -donating (2d, 2e, 2f, 2g, and 2h) groups, and besides, the reaction was even extended to a pyridyl system (2i). All the results proved that the Pd-H-MOF nanosheets can be a good candidate for reduction of nitroarene reactions. The excellent catalytic performance originates from the unique structures and gas adsorption capacity of H-MOF, combined with the surfactant-free immobilized Pd nanoparticles (Fig. 6d).

## Conclusions

The foregoing results demonstrate the availability and versatility of the competitive coordination strategy for the synthesis of H-MOF nanostructures, such as 2D H-MOF nanosheets and H-MOF nanocubes. All the unique H-MOF nanostructures were evolved through an etching process tuned by a competitive ligand. Using the 2D H-MOF nanosheets as a substrate through a simple soaking method, surfactant-free Pd nanoparticles could be *in situ* formed and immobilized. This could integrate the advantages of H-MOF and surfactant-free Pd nanoparticles, utilizing the hydrogen adsorption properties and large surface area of 2D H-MOF-5 nanosheets, as well as the surfactant-free merit of Pd nanoparticles. We believe such a competitive coordination strategy could provide an opportunity to design and synthesize other various MOF nanostructures and promote the precise morphologic control of MOFs for further applications.
