# Supplementary material for: Competitive coordination strategy for the synthesis of hierarchical-pore metal–organic framework nanostructures
Source: Chem Sci. 2016 Aug 5;7(12):7101–5. doi: 10.1039/c6sc02272c (PMC5450591; doi:10.1039/c6sc02272c)
Supplement: Supplementary file 1 [file SC-007-C6SC02272C-s001.pdf]

Electronic Supplementary Material (ESI) for Chemical Science.
This journal is © The Royal Society of Chemistry 2016

Supporting Information

Competitive Coordination Strategy for the Synthesis of Hierarchical-pore Metal-organic Framework Nanostructures

Su He[a], Yifeng Chen[b], Zhicheng Zhang[a], Bing Ni[a], Wei He[b], and Xun Wang[a]

[a] Department of Chemistry, Tsinghua University, Beijing, 100084, China
E-mail: wangxun@mail.tsinghua.edu.cn
[b] School of Pharmaceutical Science, Tsinghua University, Beijing, 100084, China

EXPERIMENTAL SECTION

Synthesis of 2D hierarchical-pore MOF-5 (H-MOF-5) nanosheets. In a typical procedure, $Zn(OAc)_2 \cdot 2H_2O$ (70mg), H_2BDC (16mg), LA (20mg) and PVP K-30 (40mg) were added to DMAC (25mL) in a 50mL flask, then heated under magnetic stirring for 30 minutes at 120°C. The products were separated via centrifugation at 10000 rpm for 10 minutes and further purified with ethanol for several times.

Synthesis of hierarchical-pore IRMOF-3 (H-IRMOF-3) nanocubes. In a typical procedure, $Zn(OAc)_2 \cdot 2H_2O$ (70mg), 2-aminoterephthalic acid (9mg), LA (20mg) and PVP K-30 (40mg) were added to DMAC (25mL) in a 50mL flask, then heated under magnetic stirring for 1 hour at 60°C. The products were separated via centrifugation at 10000 rpm for 10 minutes and further purified with ethanol for several times.

Synthesis of 2D hierarchical-pore IRMOF-8 (H-IRMOF-8) nanosheets. In a typical procedure, $Zn(OAc)_2 \cdot 2H_2O$ (22mg), 2,6-naphthalenedicarboxylic acid (11mg), LA (20mg) and PVP K-30 (40mg) were added to DMAC (25mL) in a 50mL flask, then heated under magnetic stirring for 30 minutes at 60°C. The products were separated via centrifugation at 10000 rpm for 10 minutes and further purified with ethanol for several times.

Preparation of 2D hierarchical-pore MOF-5 nanosheets immobilized Pd nanoparticles (Pd-H-MOF-5, catalyst A). Pd-H-MOF-5 catalysts (catalyst A) were prepared by soaking as-prepared H-MOF-5 nanosheets with $PdCl_2$ in DMAC-ethanol mixture (5mL, v/v=3:2). The mixture was sonicated for 5 min and then left undisturbed overnight at room temperature. The resultant solution was centrifuged at 10000 rpm for 10 min and further washed twice with ethanol to remove free Pd nanoparticles. The precipitate was redispersed in ethanol for the catalytic reaction. The loading weight of Pd in H-MOF-5 nanosheets is 7.3 wt%, as confirmed by inductively coupled plasma optical emission spectrometry (ICP-OES).

Preparation of Pd-H-MOF nanosheets post-treated with water to destroy the MOF nanostructures (catalyst B). Catalyst B was prepared by adding water to as-synthesized Pd-H-MOF-5 catalysts. The mixture was sonicated for 5 min and then left undisturbed overnight at room temperature. The resultant solution was centrifuged at 10000 rpm for 10 min and further washed twice with ethanol to remove free Pd nanoparticles. The precipitate was redispersed in ethanol for the catalytic reaction. The loading weight of Pd in catalyst B is 8.7 wt%, as confirmed by ICP-OES.

Preparation of Pd-H-MOF nanosheets with surfactant (catalyst C). Catalyst C was prepared by adding $PdCl_2$ to the previous MOF system during the reaction. $Zn(OAc)_2 \cdot 2H_2O$ (70mg), H_2BDC (16mg), LA (20mg) and PVP K-30 (40mg) were added to DMAC (25mL) in a 50mL flask and heated under magnetic stirring for 15 min at 120°C. Then 5 mg $PdCl_2$ was added to the reaction system and heated for another 15 min. The products were separated via centrifugation at 10000 rpm for 10 minutes and further purified with ethanol for several times. The precipitate was redispersed in ethanol for the catalytic reaction. The loading weight of Pd in catalyst C is 5.0 wt%,

as confirmed by ICP-OES.

Preparation of bulk MOF-5 immobilized with Pd nanoparticles (catalyst D). Firstly, bulk MOF-5 was prepared according to previous report[1]. Catalyst D was then prepared by soaking as-prepared bulk MOF-5 with $PdCl_2$ in DMAC-ethanol mixture (5mL, v/v=3:2). The mixture was sonicated for 5 min and then left undisturbed overnight at room temperature. The products were separated via centrifugation at 10000 rpm for 10 minutes and further purified with ethanol for several times. The precipitate was redispersed in ethanol for the catalytic reaction. The loading weight of Pd in catalyst C is 5.2 wt%, as confirmed by ICP-OES.

General procedures for reduction of nitroarenes: Nitro containing compound (0.5 mmol) and Pd catalyst dispersed in ethanol (2 mL, 0.01 equiv) were added to an oven-dried Schlenk tube, and the system was evacuated and refilled with H_2 (balloon) for three times. The reaction was assumed to start when the reactor was introduced into the oil bath at 60°C and vigorously stirred (ca. 1000 rpm). After 4 h, the resultant mixture was extracted with ethyl acetate (3 x 5 mL). The combined organic layer was analyzed by using GC-MS, and concentrated under reduced pressure. The crude products were further purified by flash column chromatography on silica gel to afford the desired products. The identity of the products was confirmed by comparison with literature spectroscopic data.

Characterization. Transmission electron microscopy (TEM) was conducted on a HITACHI H-7700 TEM at 100 kV, and a FEI Tecnai G^2 F20 S-Twin high-resolution (HR) TEM (HRTEM) with an accelerating voltage of 200 kV. The high-angle annular dark-field scanning TEM (HAADF-STEM), energy dispersive X-ray spectrometer (EDX) elemental mapping were performed on a FEI Tecnai G^2 F20 S-Twin HRTEM operating at 200 kV. The scanning electron microscope (SEM) was characterized by a HITACHI SU-8010. Powder X-ray diffraction (PXRD) patterns of the dried samples were recorded on a Bruker D8-advance X-ray powder diffractometer operated at 40 kV voltage and 40 mA current with CuKα radiation (λ=1.5406 Å). X-ray photoelectron spectroscopy (XPS) was carried out using a PHI Quantera SXM instrument equipped with an Al X-ray excitation source (1486.6eV). Binding energies were corrected by reference to the C 1s peak at 284.8 eV. Fourier-transformed infrared resonance (FT-IR) spectra were performed in transmission mode on a Perkin-Elmer Spectrum 100 spectrometer (Waltham, MA, USA). Thermogravimetric (TG) analysis proceeded at a constant heating rate of 10°C min^{-1} from room temperature to 800°C, on a TA-Q5000IR thermal analyzer. Brunauer-Emmett-Teller (BET) specific surface area and pore size distribution were carried out using a Quadrasorb SI-MP instrument, after the samples were degassed at 100°C for 6 h. The mesopore size distribution was calculated from BJH method using the desorption branch. Atomic force microscopy (AFM) was performed on Bruker dimension ICON. Elemental analysis of Pd and Zn in the solid samples was identified by ICP-OES (IRIS Intrepid Ⅱ XSP, ThermoFisher). ^{1}H-NMR were recorded on a Bruker Ascend™ 400 spectrometers. Chemical shifts (in ppm) were referenced to internal solvent peaks (^{1}H) or an external TMS (10% in $CDCl_3$). At the end of the catalytic run the reaction mixture was subjected to ISQ GC-MS with a ECD detector (ThermoTrace GC Ultra) using a capillary column (TR-5MS, from Thermo Scientific, length 30 m, i.d. 0.25 mm, film 0.25 μm), and the extent of conversion was calculated on the basis of the ratio of areas of starting material and product as an external standard.

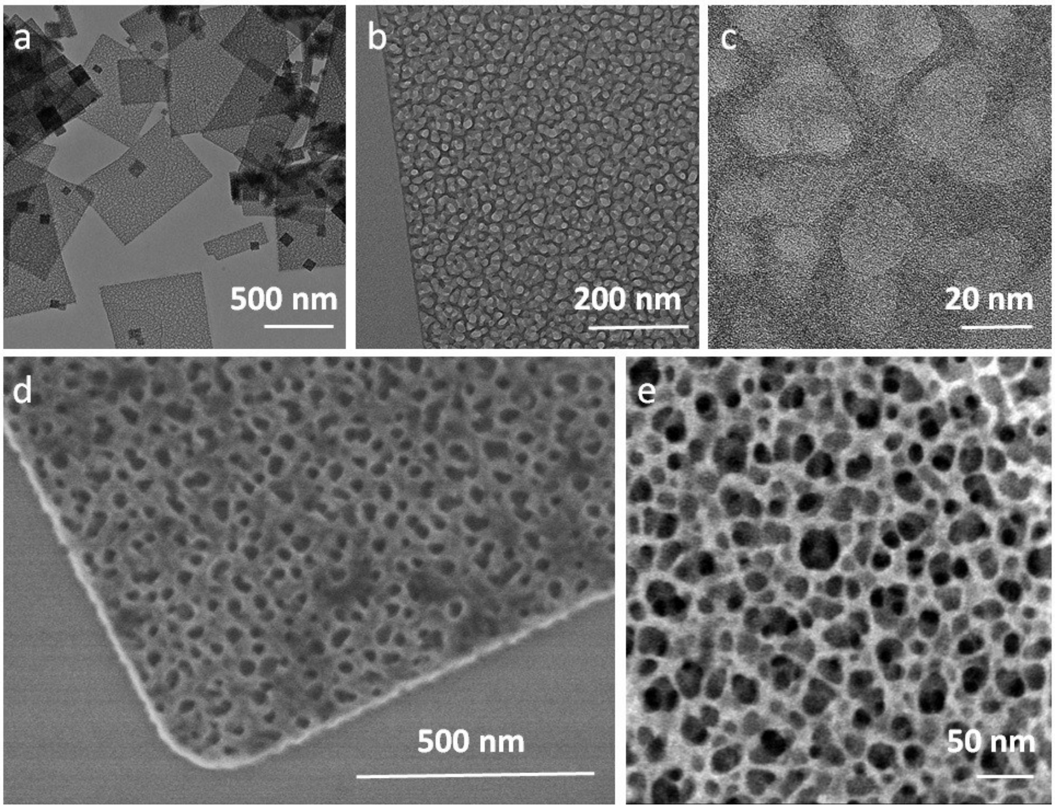

Figure S1. a,b) TEM, c) HRTEM, d) SEM and e) HAADF-STEM images of as-prepared 2D H-MOF-5 nanosheets.

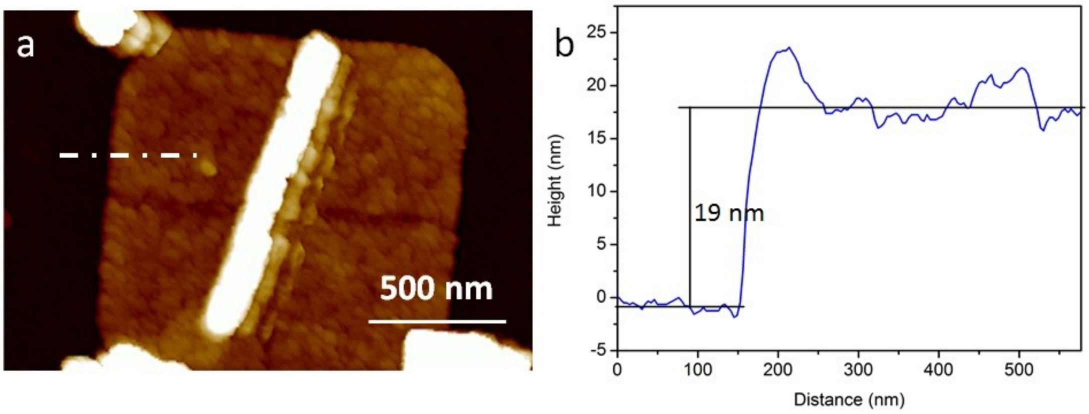

Figure S2. AFM image and corresponding height profile of as-prepared 2D H-MOF-5 nanosheets. The height profile was measured along the corresponding track shown in (a).

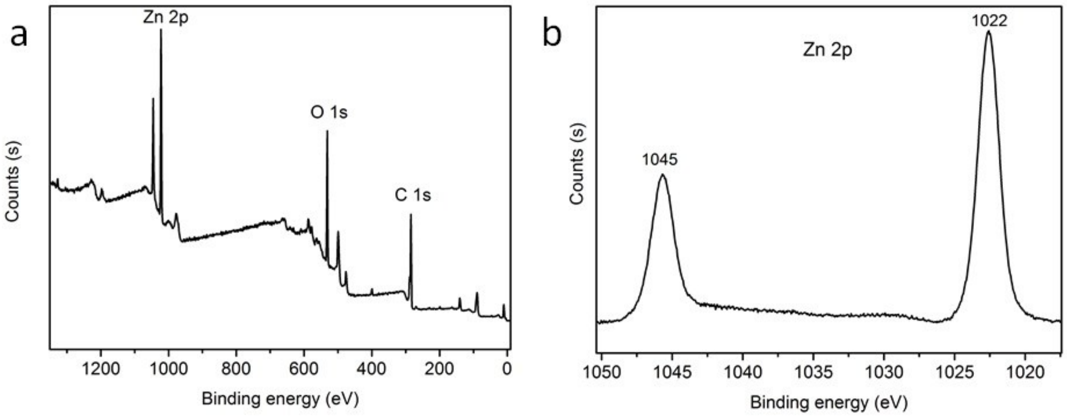

Figure S3. XPS spectra of 2D H-MOF-5 nanosheets: a) The survey spectrum; b) XPS Zn 2p spectrum. One small N 1s peak appears at 399.5 eV, which could be attributed to the residues of solvent molecule within the pores and pyrrolidone rings of PVP. The Zn 2p peaks at ~1022 eV and ~1045 eV are assigned to Zn $2p_{3/2}$ and Zn $2p_{1/2}$ for zinc(II) oxide.[2]

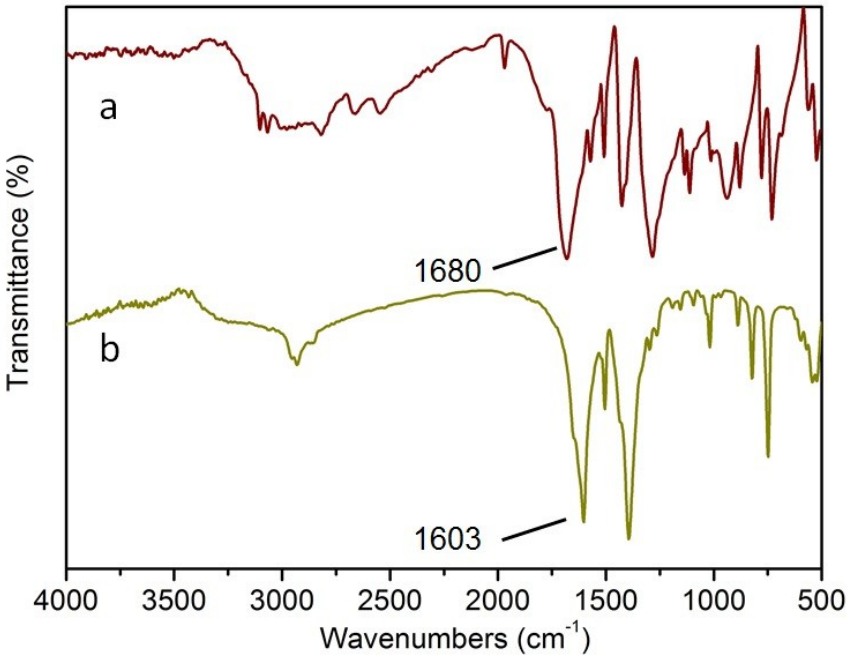

Figure S4. FT-IR spectra of a) H_2BDC and b) 2D H-MOF-5 nanosheets.

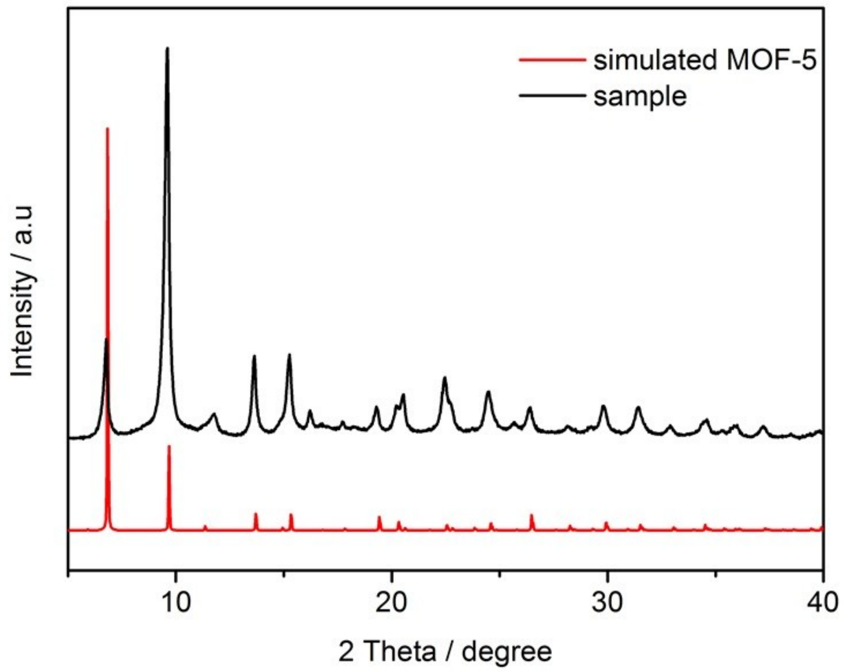

Figure S5. Typical PXRD pattern of as-prepared 2D H-MOF-5 nanosheets. The sample was degassed at 100°C under vacuum for 6 h.

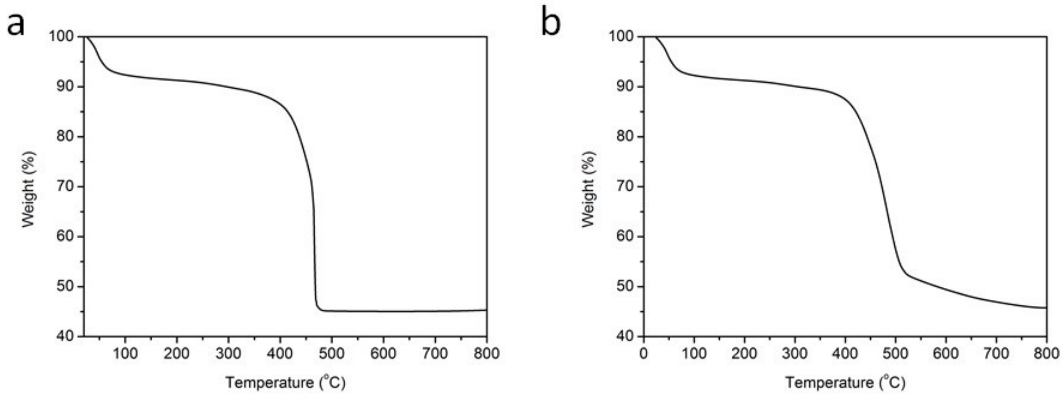

Figure S6. TG curved taken in a) air, and b) nitrogen of 2D H-MOF-5 nanosheets.

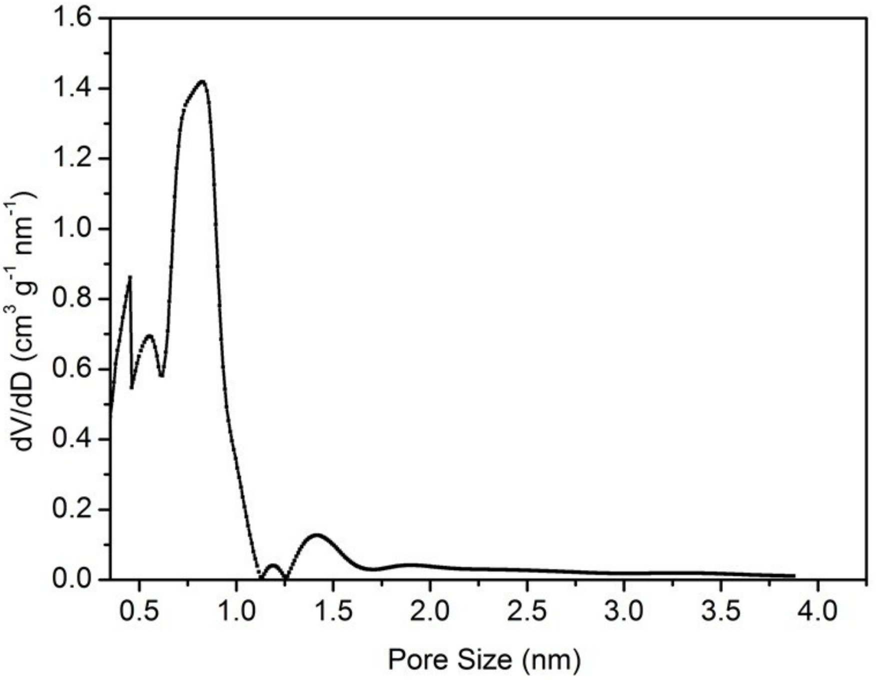

Figure S7. The micropore diameter distribution of 2D H-MOF-5 nanosheets calculated using the SF method.

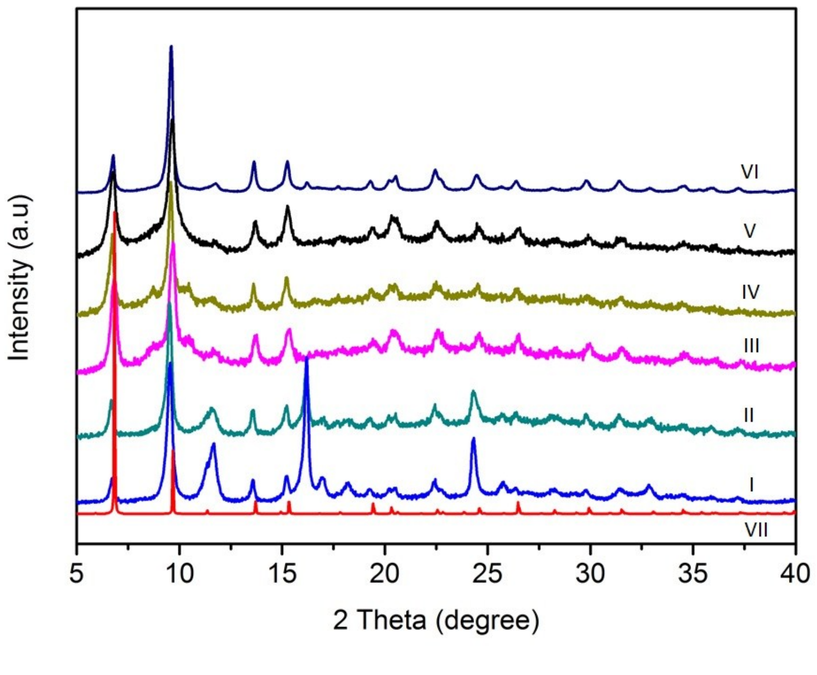

Figure S8. PXRD patterns of the product collected at different reaction stage: I) when temperature was increased to 20°C, II) when temperature was increased to 50°C, III) when temperature was increased to 80°C, IV) when temperature was increased to 100°C, V) when temperature was increased to 120°C, VI) final product and VII) simulated MOF-5. All the products were degassed at 100°C under vacuum for 6 h. The extra peaks in (I) could be the reactants that did not totally dissolve at the initial stage, with the extra highest peak corresponding to PXRD pattern of LA.

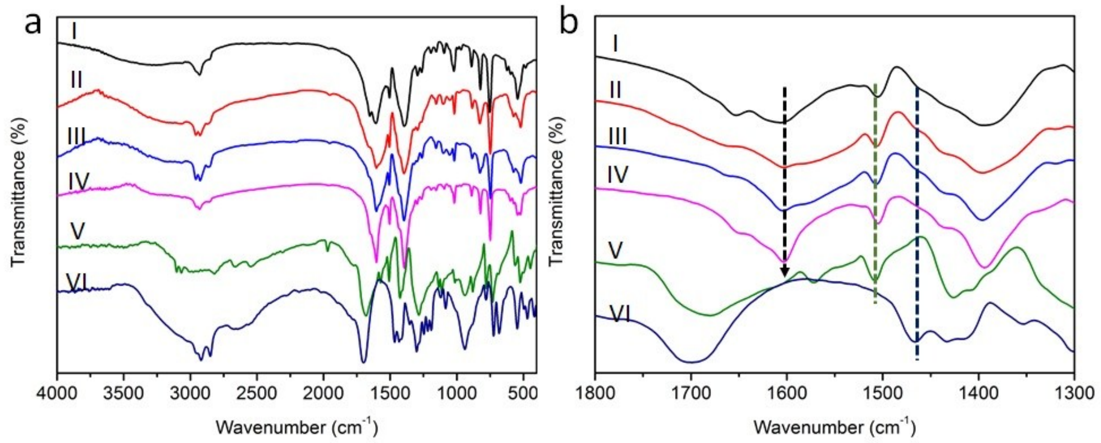

Figure S9. FT-IR spectra of the product collected at different reaction stage: I) when temperature was increased to 20^oC, II) when temperature was increased to 80^oC, III) when temperature was increased to 120^oC, IV) final product, and V) H_2BDC, and VI) LA. All the products were degassed at 100^oC under vacuum for 6 h. In (b), the black dashed arrow illustrates the enhancement of C=O stretching frequency, which is attributed to coordination of deprotonated H_2BDC to Zn_4O clusters. The green dashed line highlights the characteristic frequency of benzene, due to the existence of deprotonated H_2BDC. The blue dashed line corresponds to CH_2 characteristic frequency of LA. The absence of this peak might be ascribed to the predominant coordination of deprotonated H_2BDC to Zn_4O clusters, while the coordination of deprotonated LA is too weak to observe the characteristic frequency.

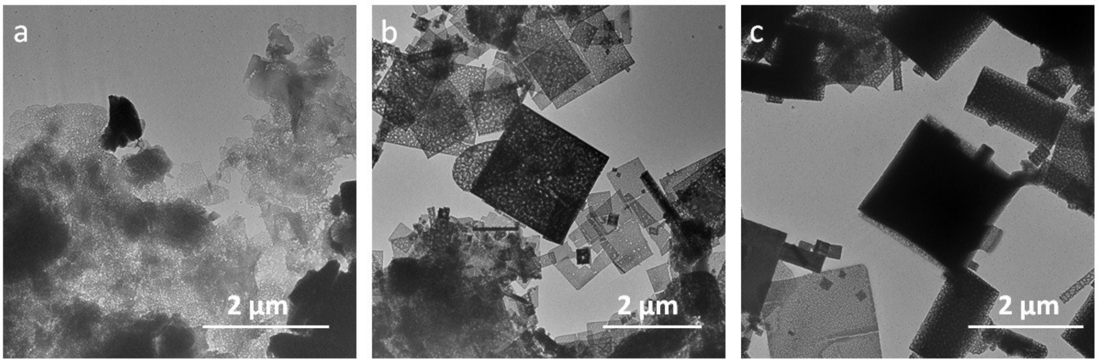

Figure S10. TEM images of the products prepared using the same procedure under different initial concentrations. a) $Zn(OAC)_2$ 22mg, H_2BDC 17mg; b) $Zn(OAC)_2$ 44mg, H_2BDC 17mg; c) $Zn(OAC)_2$ 88mg, H_2BDC 17mg.

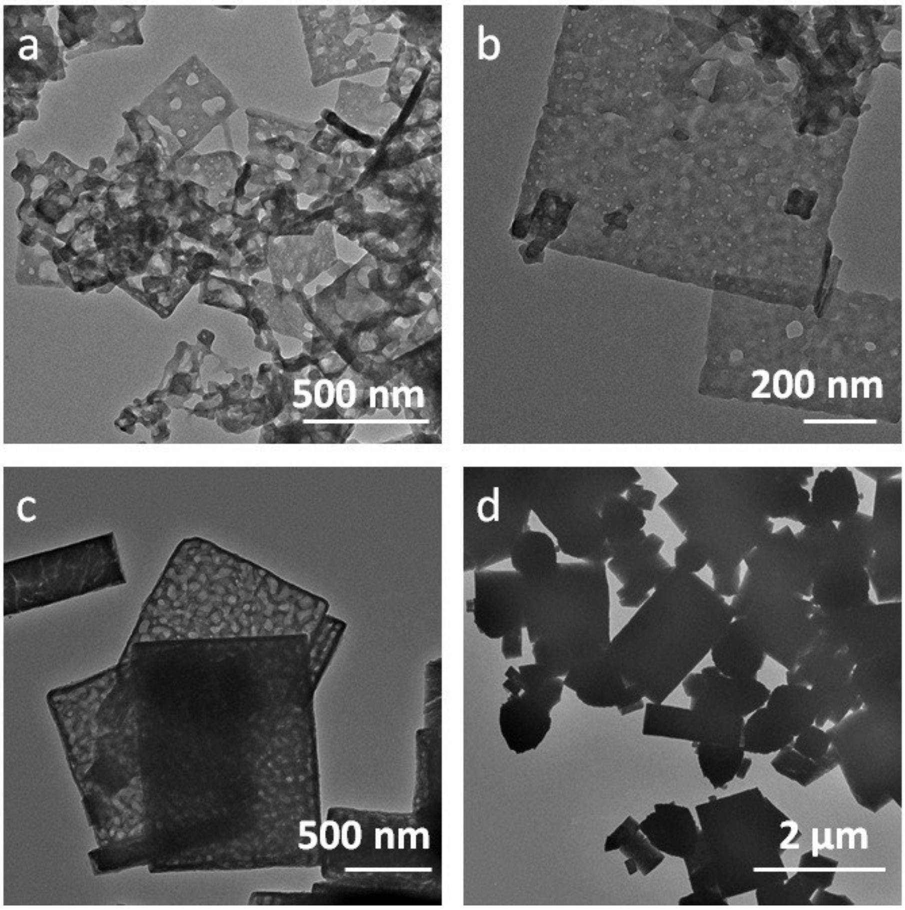

Figure S11. TEM images of the products prepared using the same procedure as 2D H-MOF-5 nanosheets but at a) 60°C, b) 80°C, c) 140°C and d) 160°C. Lower reaction temperature led to incomplete etching like (a) and (b), while higher reaction temperature resulted in thicker nanostructures like (c) and even bulk cube structures without mesopores like (d).

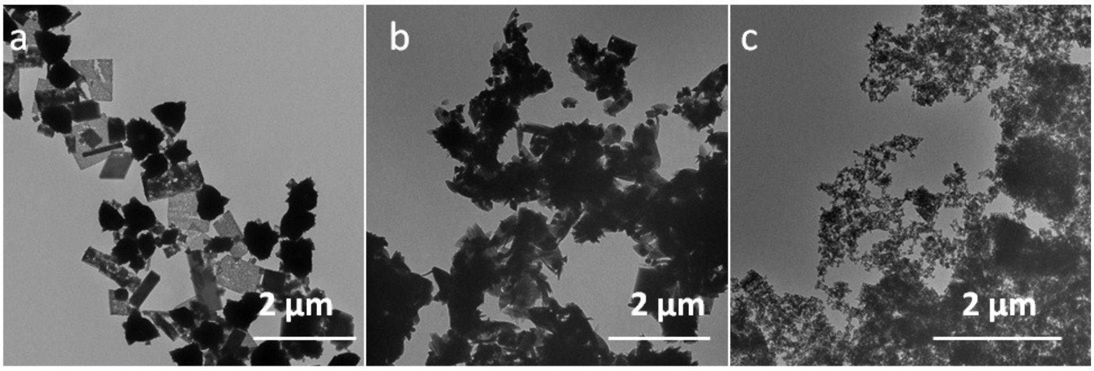

Figure S12. TEM images of the products prepared using the same procedure as 2D H-MOF-5 nanosheets but a) in the absence of LA, b) in the absence of PVP K-30 and c) using DMF instead of DMAC as solvent. Without LA, only solid nanosheets formed, indicating LA determined the mesoporous structure. Consistent with the mechanism, LA could act as a competitive ligand to coordinate to Zn_4O clusters in the initial stage. Due to its weak coordination, LA were liable to dissociate and migrate from the framework, providing copious sites for further etching. Meanwhile, PVP controlled the formation of nanosheets, similar with previous study[3]. DMAC played an essential role as well. Using DMF, similar with DMAC, could merely result in agglomerations.

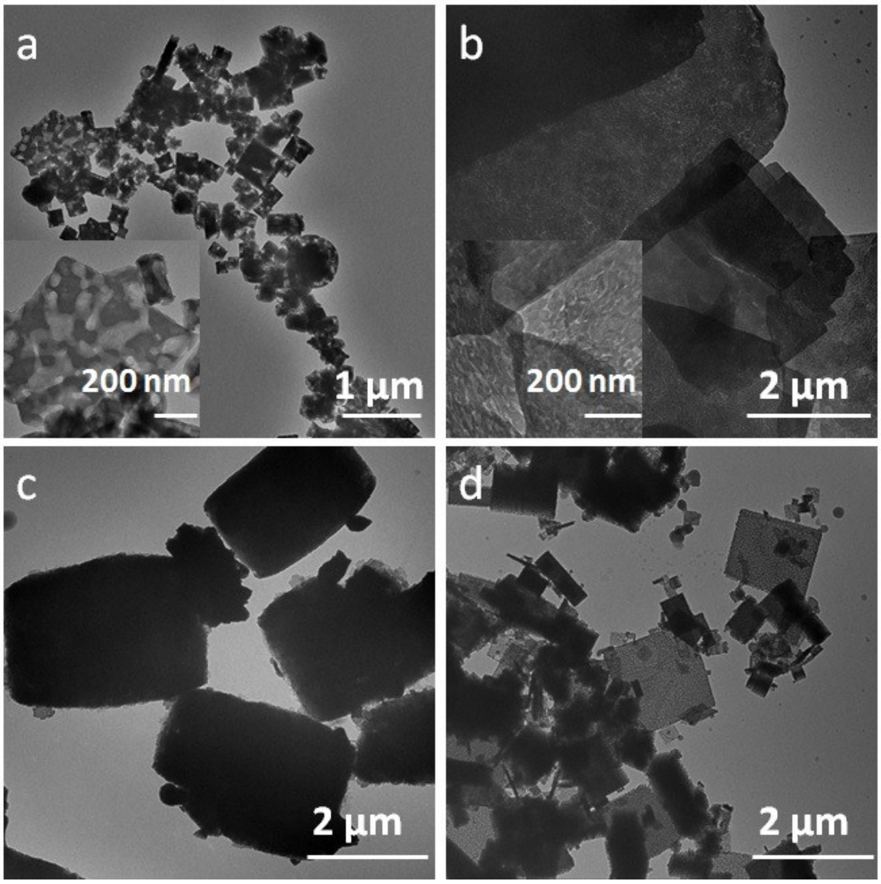

Figure S13. TEM images of the products prepared using the same procedure as 2D H-MOF-5 nanosheets but replacing PVP, K-30 with a) PVP, 58000, b) PVP, K-90, c) polyethylene glycol, 6000 (PEG, 6000) and d) cetyltrimethyl ammonium bromide (CTAB). As illustrated, PVP, K-30 plays an essential role in the synthetic system and different molecular weight leads to different morphology. Other polymer surfactants or long-chain surfactants couldn't direct the H-MOF morphology.

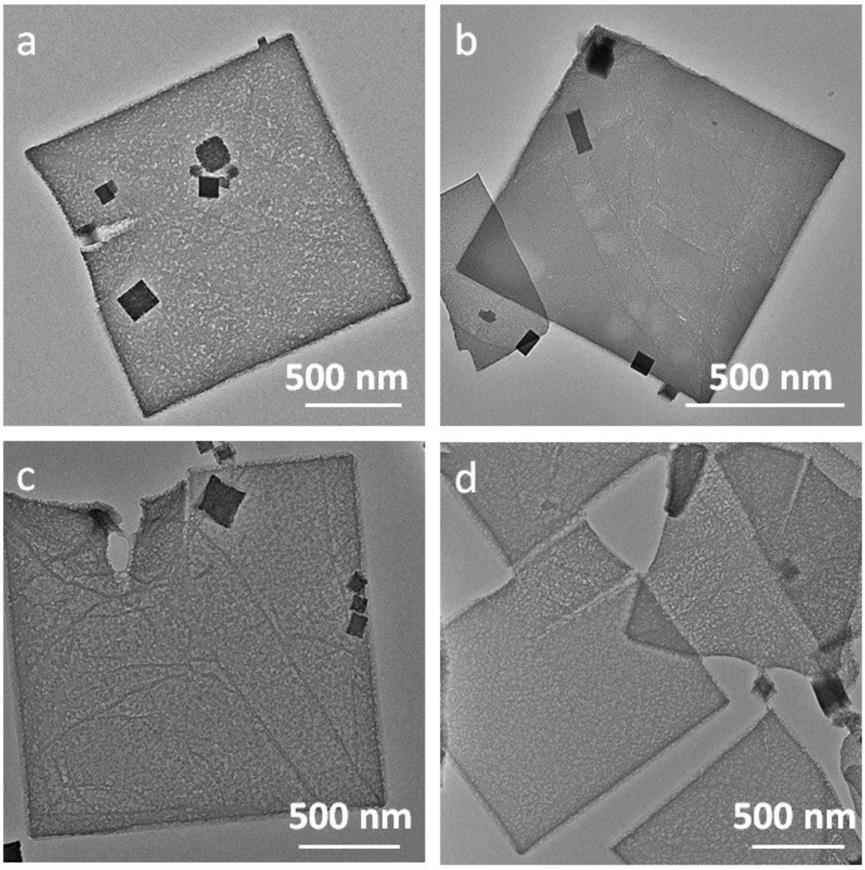

Figure S14. TEM images of the products prepared using the same procedure as 2D H-MOF-5 nanosheets but replacing LA with a) decanoic acid (DA, C-10), b) myristic acid (MA, C-14), c) palmitic acid (PA, C-16) and d) octadecanoic acid (OA, C-18). All long-chain organic acid with 10-18 carbon atoms could result in 2D H-MOF-5 nanosheets, though the mesopores of MA-directing H-MOF-5 nanosheets were relatively not so clear and smaller.

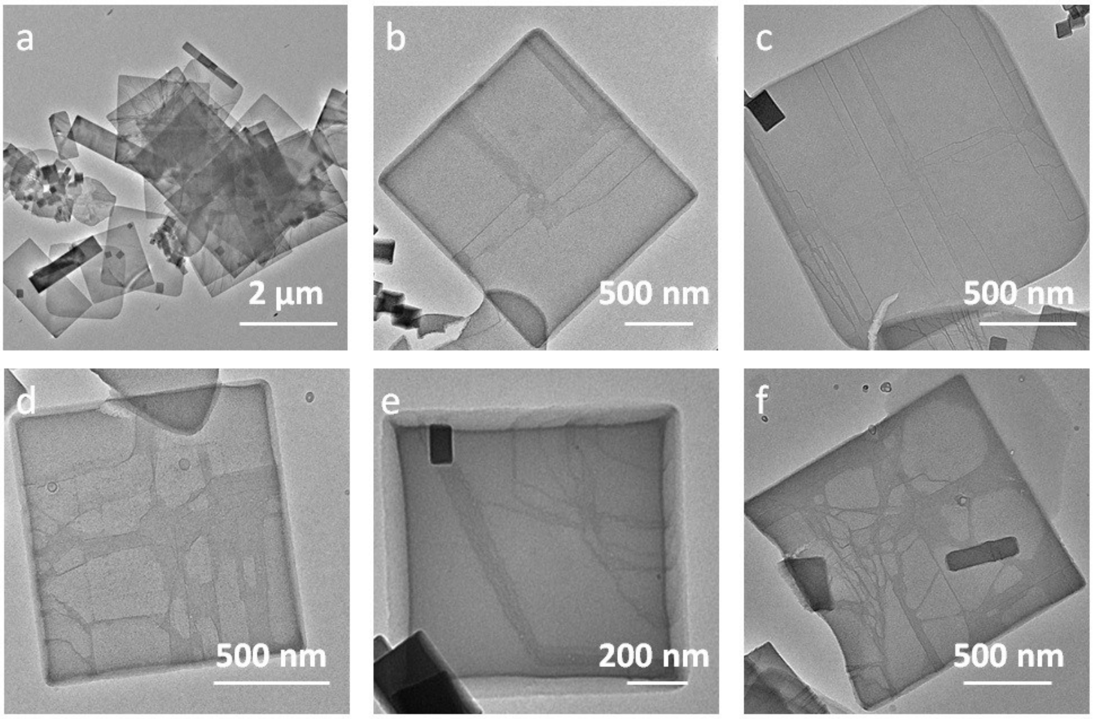

Figure S15. TEM images of the products prepared using the same procedure as 2D H-MOF-5 nanosheets except for replacing LA with a) acetic acid, b) heptanoic acid, c) benzoic acid, d) hexane, e) heptane and f) octane. All these short-chain organic acid and alkane could only generate intact nanosheets.

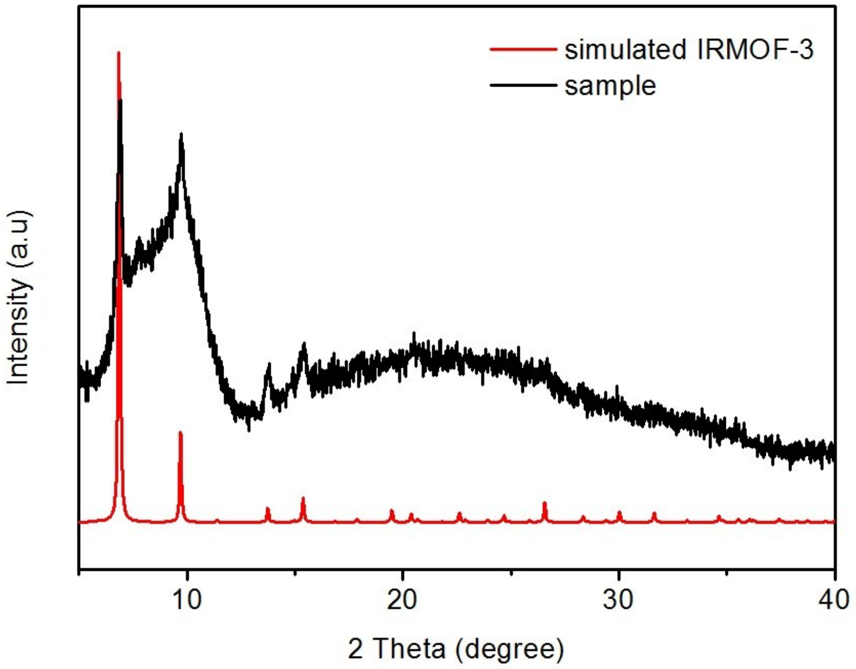

Figure S16. PXRD pattern of as prepared hierarchical-pore IRMOF-3 nanocubes. The sample was degassed at 100°C under vacuum for 6 h.

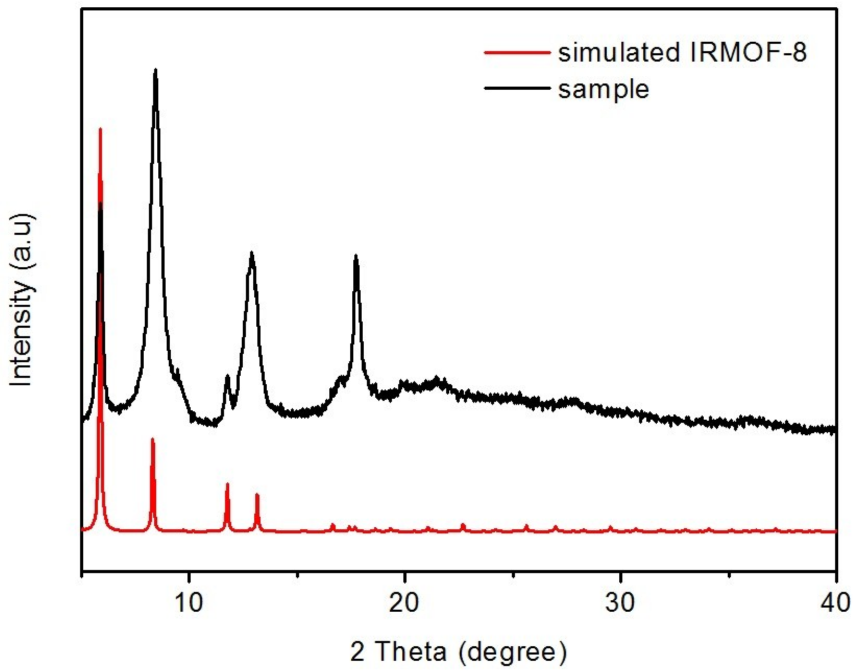

Figure S17. PXRD pattern of as prepared 2D hierarchical-pore IRMOF-8 nanosheets. The sample was degassed at 100°C under vacuum for 6 h.

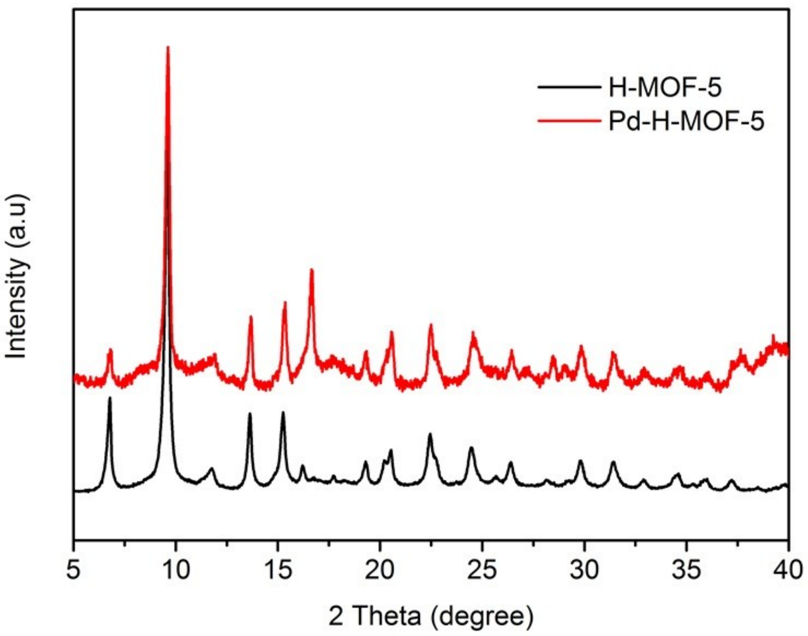

Figure S18. PXRD pattern of as prepared Pd-H-MOF-5 nanosheets. The sample was degassed at 100°C under vacuum for 6 h.

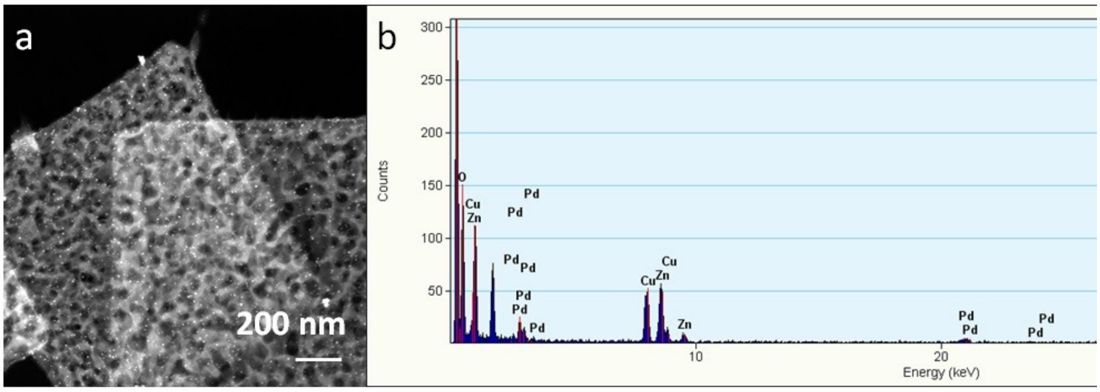

Figure S19. a) HAADF-STEM, b) EDX of the as-prepared Pd-H-MOF-5 nanosheets. In (b), the Cu peaks arise from the substrate. It is noted that the loading amount of Pd on the as-prepared Pd-H-MOF-5 nanosheets is 7.3 wt%, as determined by ICP-OES.

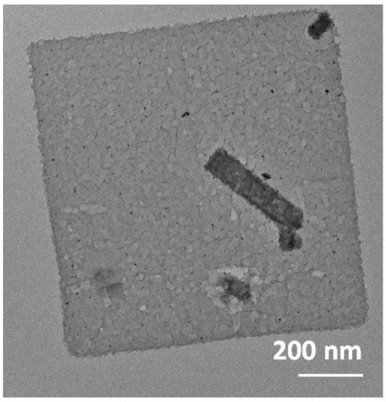

Figure S20. TEM image of the product prepared using the same procedure as 2D Pd-H-MOF-5 nanosheets, except for using $Pd(acac)_2$ instead of $PdCl_2$ as Pd^{2+} precusor.

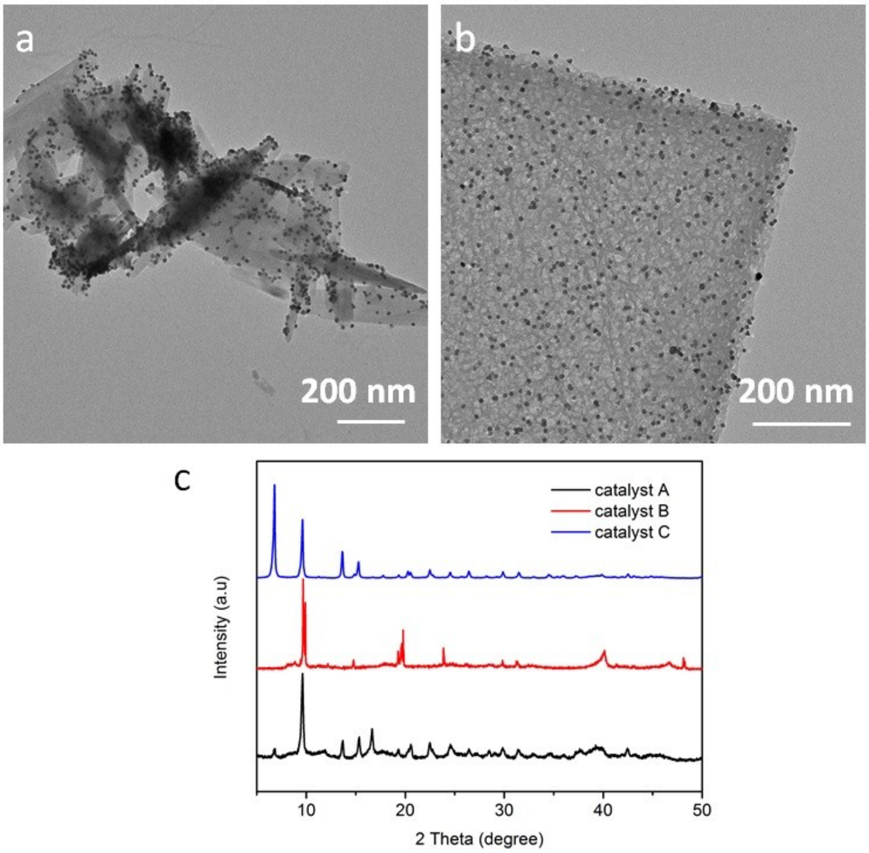

Figure S21. a,b) TEM images of catalyst B and catalyst C, respectively; c) PXRD patterns of the catalysts. The loading amounts of Pd of these two catalysts are 8.7 wt% for catalyst B and 5.0 wt% for catalyst C, as determined by ICP-OES.

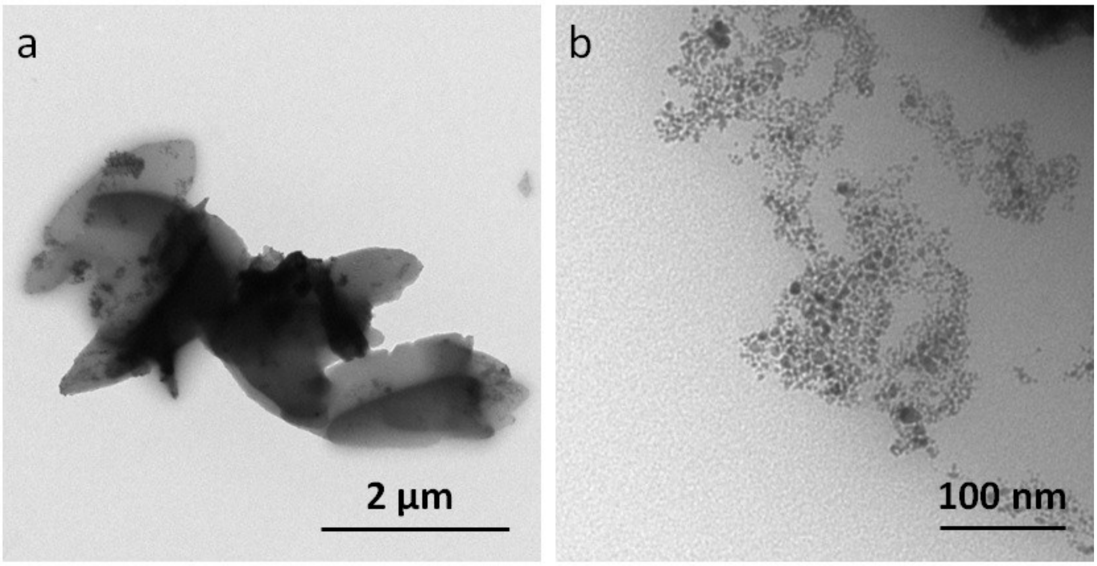

Figure S22. TEM images of catalyst A after being used for several catalytic cycles. The catalyst was recycled by centrifugation and washed by ethanol for several times, then dispersed in ethanol again. The decrease in catalytic performance also in turn testifies the structural advantage of Pd-H-MOF-5.

Comparison of other Pd catalysts reported in literature versus Pd-H-MOF-5 nanosheets for the reduction of nitrobenzene

We compared Pd-H-MOF-5 nanosheets with other Pd catalysts reported in the literature for the reduction of nitrobenzene. Though in different reaction conditions, such as temperature, reaction pressure, solvent, reactant amount and loading amount, as-prepared Pd-H-MOF-5 catalyst shows good catalytic activity (Table S1).

Table S1. Comparison of Pd catalysts for the reduction of nitrobenzene

catalyst	T (K)	P (atm)	t (h)	yield (%)	ref
**Pd/SiO_2[a]**	393	10	4.7	80	4
**SiO_2-BisILs[PF_6]-Pd^0[b]**	303	1	8.5	100	5
**PS-DVB-Pd[c]**	303	1	10	100	6
**Pd-H-MOF-5[d]**	333	1	1.5	100	This work

[a] 40 mL of ethanol, 10 mL of nitrobenzene, 1.0 g Pd/SiO_2 with Pd loading of 0.5 wt %. [b] 5 μmol of Pd and 17.5 mmol of substrate. [c] 20 mL of methanol, 9.72 mmol of nitrobenzene, Polymer (styrene divinyl benzene copolymer) anchored 2.58×10^{-5} mol Pd. [d] 2 mL of ethanol, 0.5 mmol of nitrobenzene, 7.2 mg Pd-H-MOF-5 with Pd loading of 7.3 wt %.

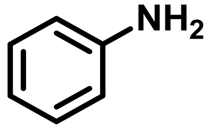

Nitrobenzene (0.5 mmol) and Pd-H-MOF-5 nanosheets dispersed in ethanol (2 mL, 0.01 equiv) were added to an oven-dried Schlenk tube, and the system was evacuated and refilled with H_2 (balloon) for three times. The reaction was assumed to start when the reactor was introduced into the oil bath at 60 ^oC and vigorously stirred (ca. 1000 rpm). After 4 h, colourless liquid was obtained as **2a** after column chromatography (eluent: petroleum ether/ethyl acetate = 10:1, 44.5mg, 95.6% yield).[7]

^1H NMR (400MHz, $CDCl_3$): 7.19 (t, 2 H), 6.78 (t, 1 H), 6.71 (dd, 2 H), 3.59 (s, 2 H). ^{13}C NMR (100 MHz, $CDCl_3$): 146.4, 129.4, 118.6, 115.2.

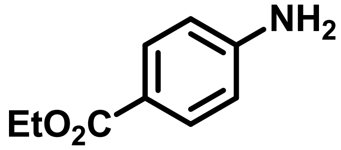

Ethyl 4-nitrobenzoate (0.5 mmol) and Pd-H-MOF-5 nanosheets dispersed in ethanol (2 mL, 0.01 equiv) were added to an oven-dried Schlenk tube, and the system was evacuated and refilled with H_2 (balloon) for three times. The reaction was assumed to start when the reactor was introduced into the oil bath at 60 ^oC and vigorously stirred (ca. 1000 rpm). After 4 h, colourless solid was obtained as **2b** after column chromatography (eluent: petroleum ether/ethyl acetate = 10:1, 70.9mg, 85.8% yield).[8]

^1H NMR (400MHz, $CDCl_3$): 7.86 (d, *J*=8.4 Hz, 2 H), 6.64 (d, *J*=8.8 Hz, 2 H), 4.32 (q, 2 H), 4.04 (s, 2 H), 1.36 (t, 3 H). ^{13}C NMR (100 MHz, $CDCl_3$): 166.9, 150.9, 131.7, 120.2, 113.9, 60.5, 14.5.

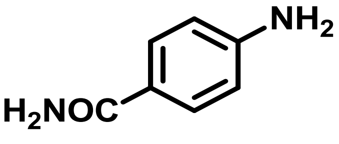

4-Nitrobenzamide (0.5 mmol) and Pd-H-MOF-5 nanosheets dispersed in ethanol (2 mL, 0.01 equiv) were added to an oven-dried Schlenk tube, and the system was evacuated and refilled with H_2 (balloon) for three times. The reaction was assumed to start when the reactor was introduced into the oil bath at 60 ^oC and vigorously stirred (ca. 1000 rpm). After 4 h, white solid was obtained as **2c** after column chromatography (eluent: petroleum ether/ethyl acetate = 1:1, 59.5mg, 87.4% yield).[9]

^1H NMR (400MHz, [D6] DMSO): 7.58 (d, *J*=8.4 Hz, 2 H), 7.48 (s, 1 H), 6.79 (s, 1H), 6.52 (d, *J*=8.4 Hz, 2 H), 5.57(s, 2 H). ^{13}C NMR (100 MHz, [D6] DMSO): 168.0, 151.6, 129.1, 121.0, 112.4.

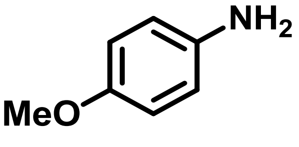

1-Methoxy-4-nitrobenzene (0.5 mmol) and Pd-H-MOF-5 nanosheets dispersed in ethanol (2 mL, 0.01 equiv) were added to an oven-dried Schlenk tube, and the system was evacuated and refilled with H_2 (balloon) for three times. The reaction was assumed to start when the reactor was introduced into the oil bath at 60 °C and vigorously stirred (ca. 1000 rpm). After 4 h, white solid was obtained as **2d** after column chromatography (eluent: petroleum ether/ethyl acetate = 10:1, 59.3mg, 96.3% yield).[7]

^{1}H-NMR (400 MHz , CDCl$_3$): 6.76 (d, *J*=8.4 Hz, 2H), 6.66 (d, *J*=8. 4 Hz, 2H), 3.75 (s, 3H), 3.33 (s, 2H). ^{13}C NMR (100 MHz, CDCl$_3$): 152.9, 140.0, 116.5, 114.9, 55.8.

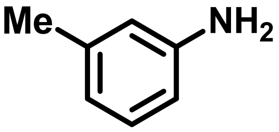

1-Methyl-3-nitrobenzene (0.5 mmol) and Pd-H-MOF-5 nanosheets dispersed in ethanol (2 mL, 0.01 equiv) were added to an oven-dried Schlenk tube, and the system was evacuated and refilled with H_2 (balloon) for three times. The reaction was assumed to start when the reactor was introduced into the oil bath at 60 °C and vigorously stirred (ca. 1000 rpm). After 4 h, colourless liquid was obtained as **2e** after column chromatography (eluent: petroleum ether/ethyl acetate = 10:1, 52.2mg, 97.4% yield).[10]

^{1}H NMR (400MHz, CDCl$_3$): 7.07 (t, 2 H), 6.62 (d, 1 H), 6.54 (dd, 2 H), 3.66 (s, 2 H), 2.29 (s, 3 H). ^{13}C NMR (100 MHz, CDCl$_3$): 146.3, 139.3, 129.3, 119.7, 116.2, 112.5, 21.5 .

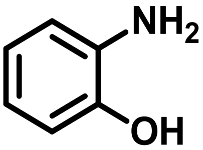

2-Nitrophenol (0.5 mmol) and Pd-H-MOF-5 nanosheets dispersed in ethanol (2 mL, 0.01 equiv) were added to an oven-dried Schlenk tube, and the system was evacuated and refilled with H_2 (balloon) for three times. The reaction was assumed to start when the reactor was introduced into the oil bath at 60 °C and vigorously stirred (ca. 1000 rpm). After 4 h, white solid was obtained as **2f** after column chromatography (eluent: petroleum ether/ethyl acetate = 1:1, 50.5mg, 92.6% yield).[8]

^{1}H NMR (400MHz, CDCl$_3$): 8.90 (s, 1 H), 6.61 (m, 3 H), 6.40 (t, 1 H), 4.44 (s, 2 H). ^{13}C NMR (100 MHz, CDCl$_3$): 143.9, 136.5, 119.5, 116.4, 1140.4, 114.3 .

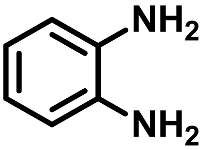

2-Nitroaniline (0.5 mmol) and Pd-H-MOF-5 nanosheets dispersed in ethanol (2 mL, 0.01 equiv) were added to an oven-dried Schlenk tube, and the system was evacuated and refilled with H_2 (balloon) for three times. The reaction was assumed to start when the reactor was introduced into the oil bath at 60 °C and vigorously stirred (ca. 1000 rpm). After 4 h, grey solid was obtained as **2g** after column chromatography (eluent: petroleum ether/ethyl acetate = 1:1, 49.5mg, 91.5%

yield).[8]

^{1}H NMR (400MHz, $CDCl_3$): 6.72 (m, 4 H), 3.33 (s, 4 H). ^{13}C NMR (100 MHz, $CDCl_3$): 134.8, 120.4, 116.8.

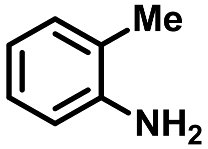

1-Methyl-2-nitrobenzene (0.5 mmol) and Pd-H-MOF-5 nanosheets dispersed in ethanol (2 mL, 0.01 equiv) were added to an oven-dried Schlenk tube, and the system was evacuated and refilled with H_2 (balloon) for three times. The reaction was assumed to start when the reactor was introduced into the oil bath at 60 °C and vigorously stirred (ca. 1000 rpm). After 4 h, yellow liquid was obtained as **2h** after column chromatography (eluent: petroleum ether/ethyl acetate = 10:1, 51.1mg, 95.4% yield).[7]

^{1}H NMR (400 MHz, $CDCl_3$): *δ* 7.06 (t , 2H), 6.74 (m, 2H), 3.73 (s, 2H), 2.20 (s, 3H). ^{13}C NMR (100 MHz, $CDCl_3$): *δ* 144.2, 130.6, 127.0, 122.7, 119.0, 115.3, 17.4.

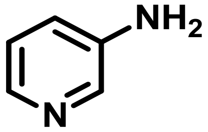

3-Nitropyridine (0.5 mmol) and Pd-H-MOF-5 nanosheets dispersed in ethanol (2 mL, 0.01 equiv) were added to an oven-dried Schlenk tube, and the system was evacuated and refilled with H_2 (balloon) for three times. The reaction was assumed to start when the reactor was introduced into the oil bath at 60 °C and vigorously stirred (ca. 1000 rpm). After 4 h, colourless liquid was obtained as **2i** after column chromatography (eluent: petroleum ether/ethyl acetate = 1:1, 39.8mg, 84.5% yield).[11]

^{1}H NMR (400MHz, $CDCl_3$): 7.92 (s, 1 H), 7.71 (s, 1 H), 6.99(m, 1 H), 6.89 (m, 1 H), 5.25 (s, 2 H). ^{13}C NMR (100 MHz, $CDCl_3$): 144.8, 136.9, 136.4, 123.5, 119.6.

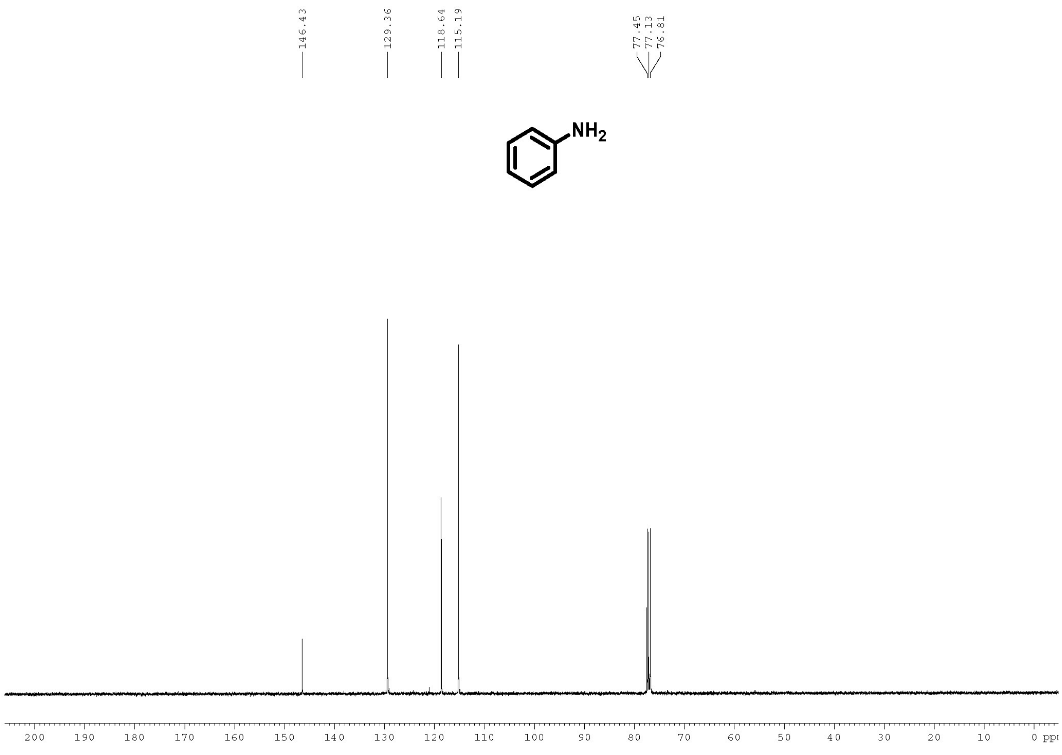
146.43
129.36
118.64
115.19
77.45
77.13
76.81
NH2
200 190 180 170 160 150 140 130 120 110 100 90 80 70 60 50 40 30 20 10 0 ppm

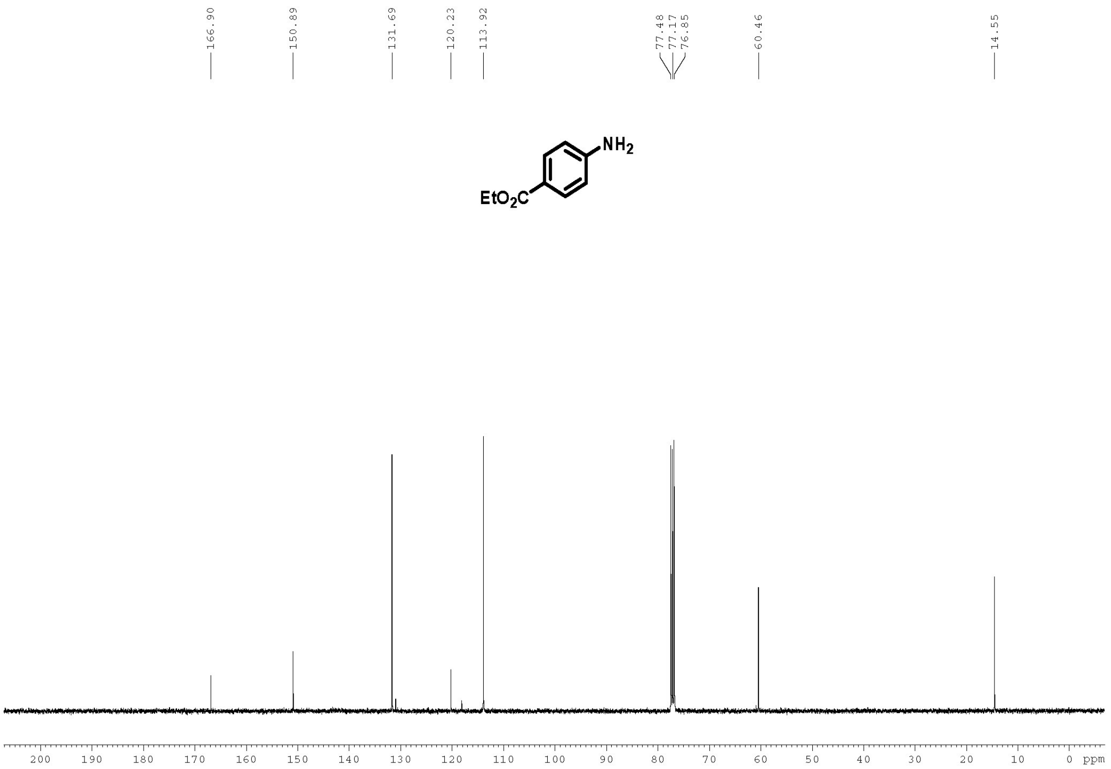
166.90
150.89
131.69
120.23
113.92
77.48
77.17
76.85
60.46
14.55
NH2
EtO2C
200 190 180 170 160 150 140 130 120 110 100 90 80 70 60 50 40 30 20 10 0 ppm

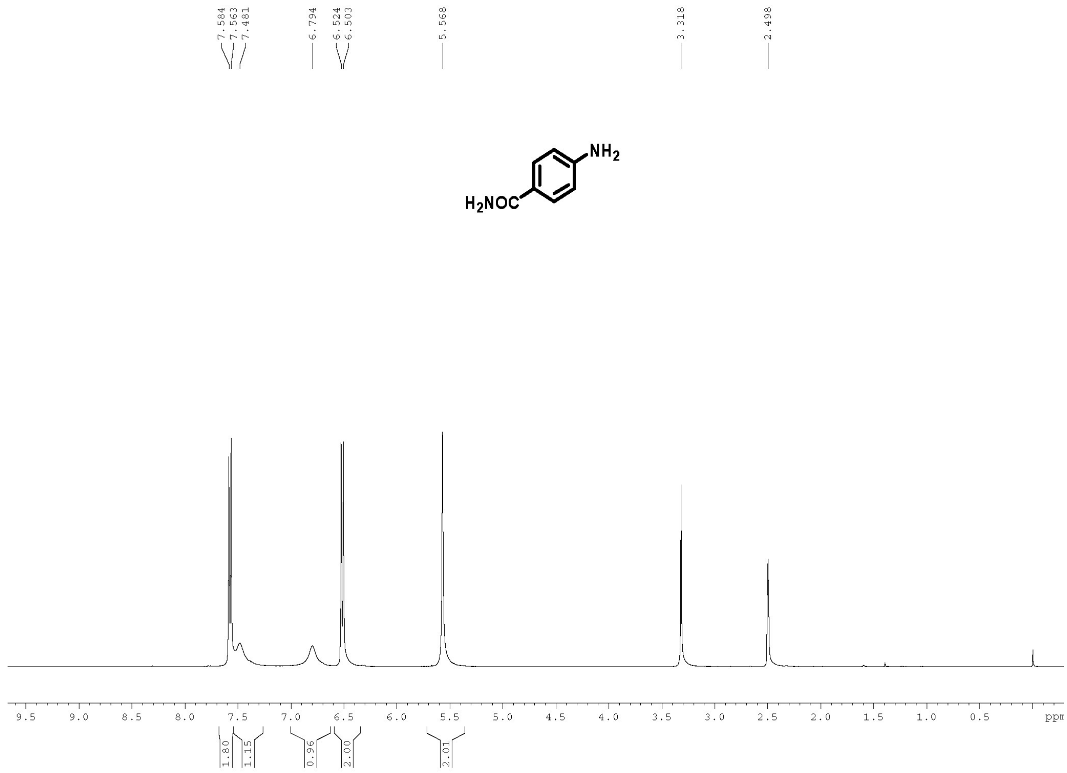
7.584
7.563
7.481
6.794
6.524
6.503
5.568
3.318
2.498
NH_2
H_2NOC
9.5 9.0 8.5 8.0 7.5 7.0 6.5 6.0 5.5 5.0 4.5 4.0 3.5 3.0 2.5 2.0 1.5 1.0 0.5 ppm
1.80
1.15
0.96
2.00
2.01

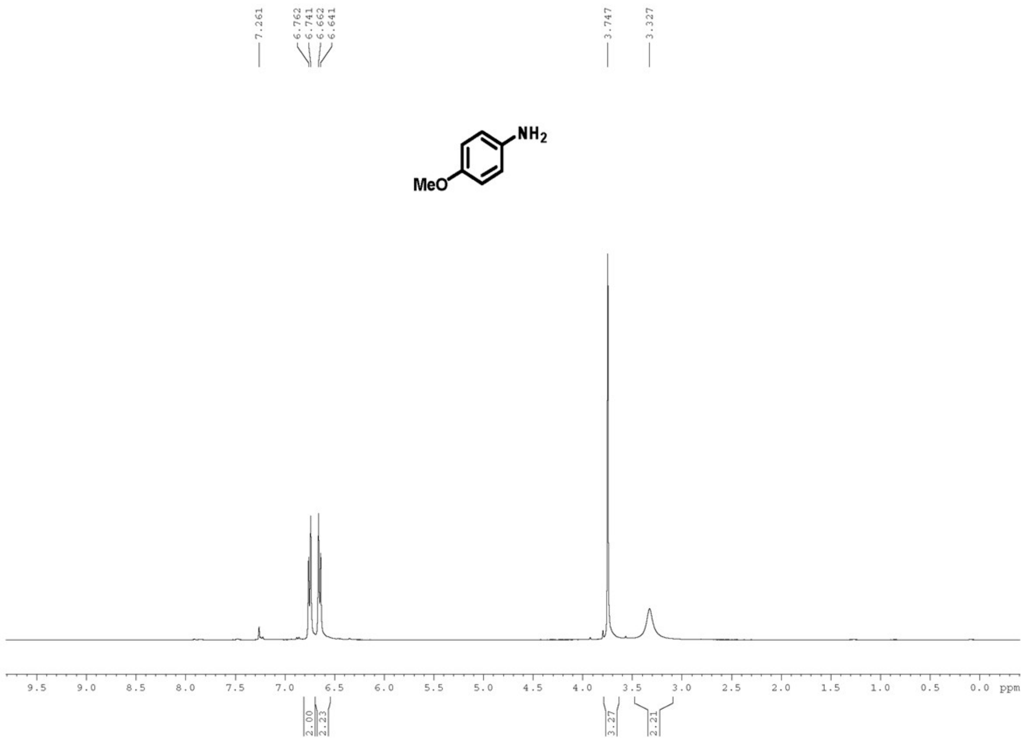
7.261
6.762
6.741
6.662
6.641
3.747
3.327
NH2
MeO
9.5 9.0 8.5 8.0 7.5 7.0 6.5 6.0 5.5 5.0 4.5 4.0 3.5 3.0 2.5 2.0 1.5 1.0 0.5 0.0 ppm
2.00
2.23
3.27
2.21

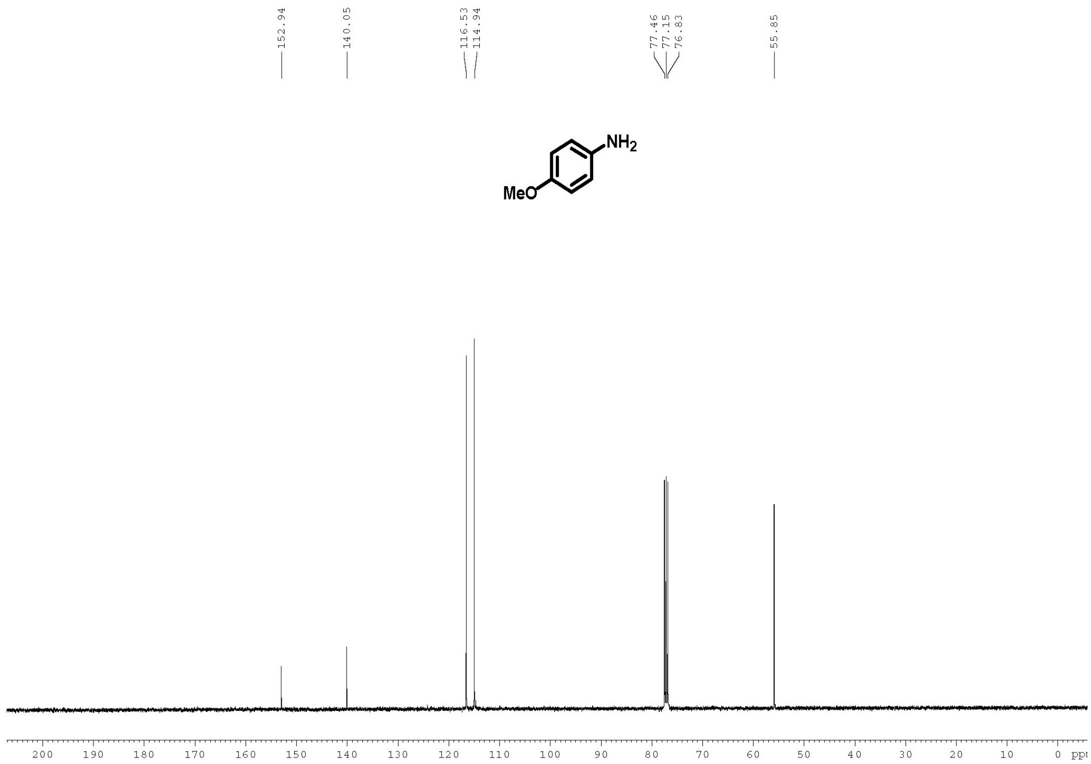
152.94
140.05
116.53
114.94
77.46
77.15
76.83
55.85
NH2
MeO
200 190 180 170 160 150 140 130 120 110 100 90 80 70 60 50 40 30 20 10 0 ppm

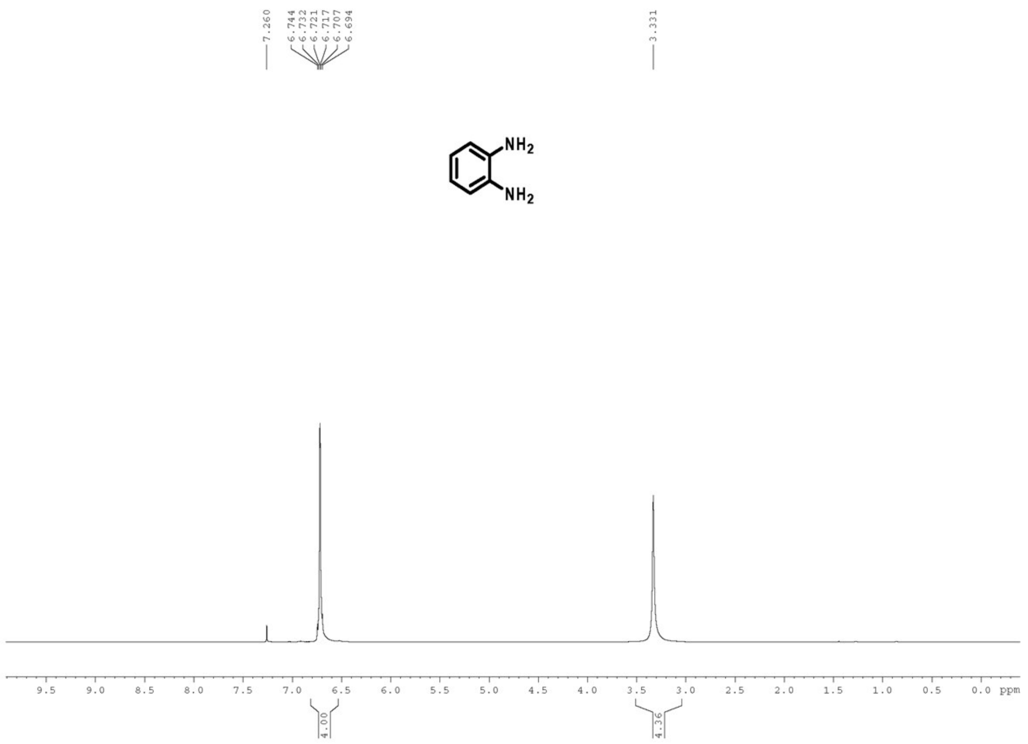
7.260
6.744
6.732
6.721
6.717
6.707
6.694
3.331
NH2
NH2
9.5 9.0 8.5 8.0 7.5 7.0 6.5 6.0 5.5 5.0 4.5 4.0 3.5 3.0 2.5 2.0 1.5 1.0 0.5 0.0 ppm
4.00
4.36

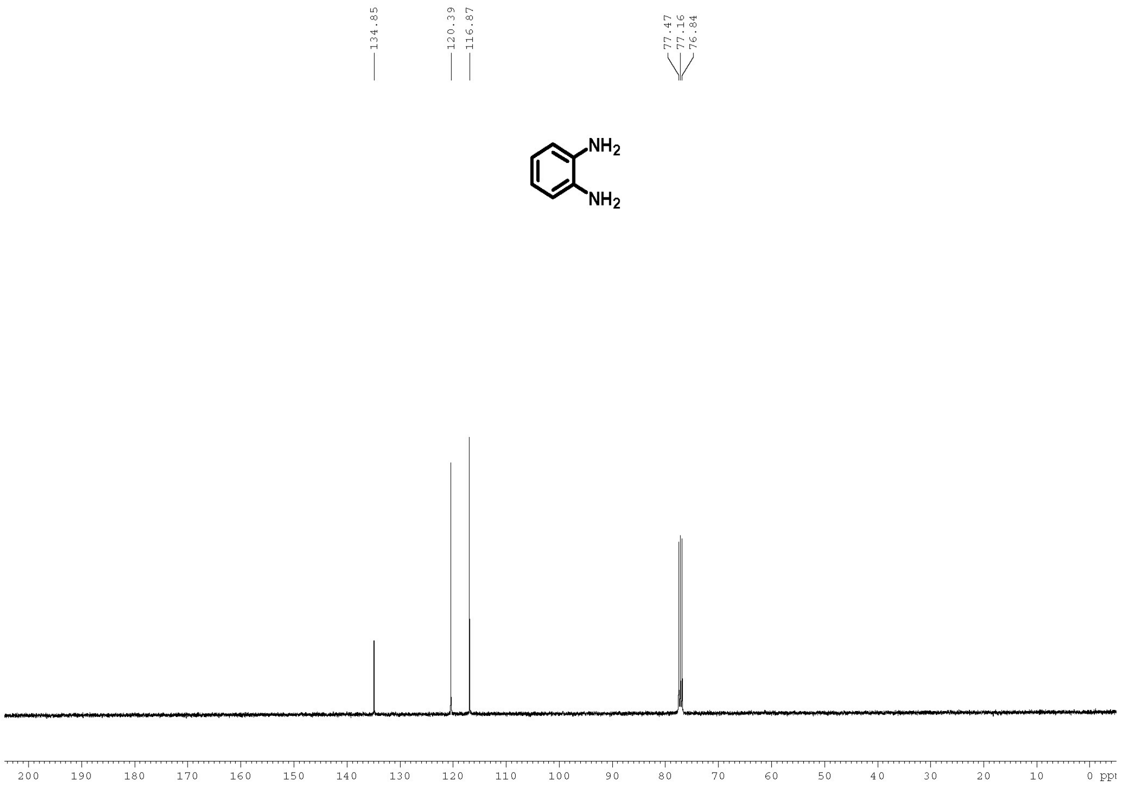
134.85
120.39
116.87
77.47
77.16
76.84
NH2
NH2
200 190 180 170 160 150 140 130 120 110 100 90 80 70 60 50 40 30 20 10 0 ppm

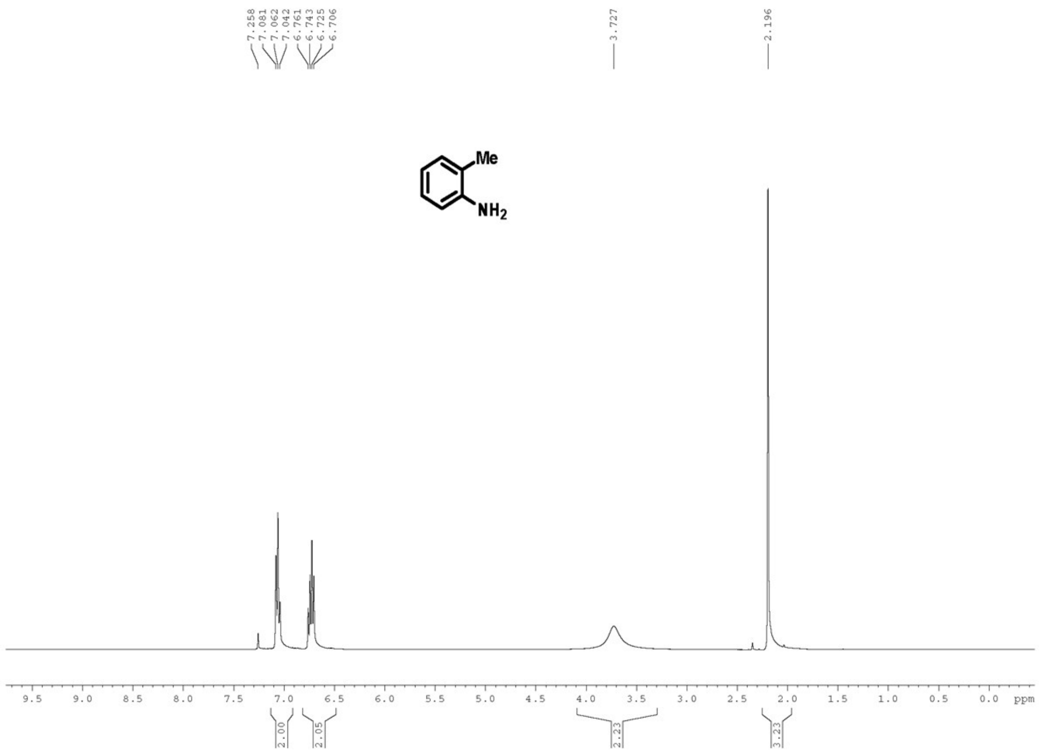
7.258
7.081
7.062
7.042
6.761
6.743
6.725
6.706
3.727
2.196
Me
NH2
9.5 9.0 8.5 8.0 7.5 7.0 6.5 6.0 5.5 5.0 4.5 4.0 3.5 3.0 2.5 2.0 1.5 1.0 0.5 0.0 ppm
2.00
2.05
2.23
3.23

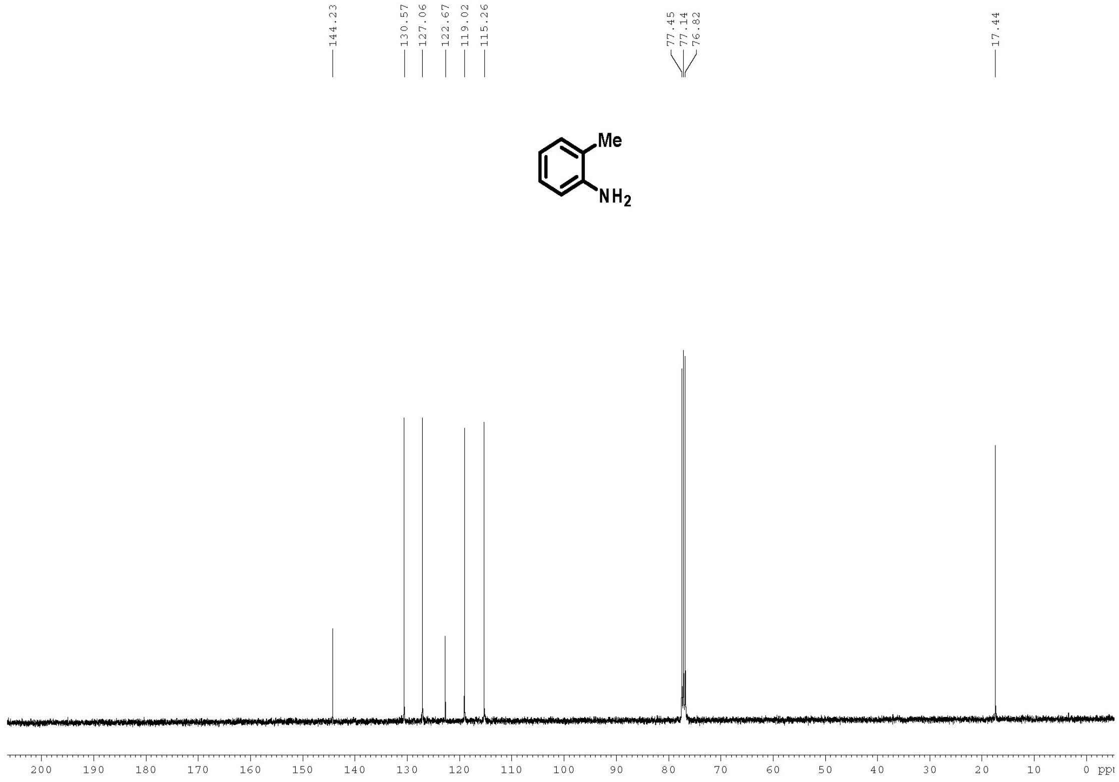
144.23
130.57
127.06
122.67
119.02
115.26
77.45
77.14
76.82
17.44
Me
NH2
200 190 180 170 160 150 140 130 120 110 100 90 80 70 60 50 40 30 20 10 0 ppm

1. J. Hafizovic, M. Bjørgen, U. Olsbye, P. D. C. Dietzel, S. Bordiga, C. Prestipino, C. Lamberti and K. P. Lillerud, *J. Am. Chem. Soc.*, 2007, **129**, 3612-3620.
2. Z. Zhang, Y. Chen, S. He, J. Zhang, X. Xu, Y. Yang, F. Nosheen, F. Saleem, W. He and X. Wang, *Angew. Chem. Int. Ed.*, 2014, **126**, 12725-12729.
3. M. Zhao, Y. Wang, Q. Ma, Y. Huang, X. Zhang, J. Ping, Z. Zhang, Q. Lu, Y. Yu, H. Xu, Y. Zhao and H. Zhang, *Adv. Mater.*, 2015, **27**, 7372-7378.
4. X. Yu, M. Wang and H. Li, *Appl. Catal. A: General*, 2000, **202**, 17-22.
5. J. Li, X.-Y. Shi, Y.-Y. Bi, J.-F. Wei and Z.-G. Chen, *ACS Catalysis*, 2011, **1**, 657-664.
6. D. R. Patel and R. N. Ram, *J. Mol. Catal. A: Chem.l*, 1998, **130**, 57-64.
7. S. Ahammed, A. Saha and B. C. Ranu, *The Journal of Organic Chemistry*, 2011, **76**, 7235-7239.
8. Z. Zhao, H. Yang, Y. Li and X. Guo, *Green Chemistry*, 2014, **16**, 1274-1281.
9. S. Sharma, M. Kumar, V. Kumar and N. Kumar, *The Journal of Organic Chemistry*, 2014, **79**, 9433-9439.
10. A. Saha and B. Ranu, *The Journal of Organic Chemistry*, 2008, **73**, 6867-6870.
11. R. V. Jagadeesh, D. Banerjee, P. B. Arockiam, H. Junge, K. Junge, M.-M. Pohl, J. Radnik, A. Bruckner and M. Beller, *Green Chemistry*, 2015, **17**, 898-902.
